# BCA2/Rabring7 Targets HIV-1 Gag for Lysosomal Degradation in a Tetherin-Independent Manner

**DOI:** 10.1371/journal.ppat.1004151

**Published:** 2014-05-22

**Authors:** Ramya Nityanandam, Ruth Serra-Moreno

**Affiliations:** Division of Immunology, New England Primate Research Center, Harvard Medical School, Southborough, Massachusetts, United States of America; Universitätklinikum Heidelberg, Germany

## Abstract

BCA2 (Rabring7, RNF115 or ZNF364) is a RING-finger E3 ubiquitin ligase that was identified as a co-factor in the restriction imposed by tetherin/BST2 on HIV-1. Contrary to the current model, in which BCA2 lacks antiviral activity in the absence of tetherin, we found that BCA2 possesses tetherin-independent antiviral activity. Here we show that the N-terminus of BCA2 physically interacts with the Matrix region of HIV-1 and other retroviral Gag proteins and promotes their ubiquitination, redistribution to endo-lysosomal compartments and, ultimately, lysosomal degradation. The targeted depletion of BCA2 in tetherin-expressing and tetherin-deficient cells results in a significant increase in virus release and replication, indicating that endogenous BCA2 possesses antiviral activity. Therefore, these results indicate that BCA2 functions as an antiviral factor that targets HIV-1 Gag for degradation, impairing virus assembly and release.

## Author Summary

Tetherin (also known as BST2, CD317 or HM1.24) is an interferon-inducible cellular factor that interferes with the release of enveloped viruses from infected cells. A recent study identified BCA2 (Breast Cancer-Associated gene 2, also known as RNF115, ZNF364 or Rabring7), a RING-finger E3 ubiquitin ligase, as a co-factor in the tetherin-mediated restriction of HIV-1. According to this model, BCA2 interacts with sequences in the N-terminus of tetherin to promote the internalization and lysosomal degradation of tethered HIV-1 particles, with no apparent antiviral activity in cells not expressing tetherin. However, here we show for the first time that BCA2 inhibits virus production for HIV-1 and other retroviruses in a tetherin-independent manner by reducing the cellular levels of Gag – the precursor of the structural proteins Matrix, Capsid, Nucleocapsid and p6. Hence, contrary to its reported role as a tetherin co-factor, BCA2 functions as a tetherin-independent antiviral factor that impairs virus assembly and release.

## Introduction

HIV-1, as well as other animal viruses, relies on the contributions of cellular molecules to ensure virus replication [1]. Conversely, mammalian cells have evolved mechanisms to block different stages of the virus life-cycle, thereby suppressing virus production. These proteins are generally known as restriction factors, and they provide an early antiviral defense. Despite their antiviral activity, the primate lentiviruses have developed mechanisms to circumvent these cellular factors. The need to escape this first line of defense seems to be an important driving force behind the acquisition of these countermeasures, indicating that overcoming these proteins is important for virus replication and infectivity [2], [3], [4].

So far, five potent restriction factors have been shown to effectively block HIV and SIV replication: APOBEC3 members (A3), TRIM5 proteins, Mx2/MxB, SAMHD1 and tetherin/BST2 [3], [5], [6], [7], [8], [9], [10], [11], [12], [13], [14], [15], [16], [17], [18], [19]. Tetherin, an interferon-inducible transmembrane protein, impedes the release of enveloped viruses from infected cells, by trapping nascent virions to the cell surface [15], [16], [20]. However, the primate lentiviruses have evolved different mechanisms to antagonize tetherin. Whereas HIV-1 Vpu and HIV-2 Env counteract human tetherin [15], [16], [21], we and others identified Nef as the protein used by most SIVs to overcome non-human primate tetherin, and we further characterized the mechanism of tetherin antagonism by Nef [22], [23], [24], [25], [26].

A recent study [27], identified BCA2 (Breast Cancer-Associated gene 2) –a RING-finger E3 ubiquitin ligase– as a co-factor in the restriction imposed by tetherin on HIV-1, lacking antiviral activity in cells not expressing tetherin. Miyakawa and collaborators showed that BCA2 interacts with the cytoplasmic domain of tetherin to facilitate the internalization and lysosomal degradation of tethered virions, and that the E3 ligase activity of BCA2 is dispensable to enhance tetherin-mediated restriction [27]. Contrary to this model, we found that BCA2 is also a tetherin-independent antiviral factor. In particular, BCA2 reduces the cellular levels of HIV-1 and other retroviral Gag proteins. In contrast to the tetherin-dependent activity of BCA2, an intact RING-finger domain is required for tetherin-independent restriction. Consistent with this observation, the HIV-1 Gag protein, as well as other retroviral Gag proteins, become ubiquitinated in cells expressing BCA2. Immunoprecipitation assays showed that the N-terminus of BCA2 interacts with HIV-1 Gag through its Matrix region, and that mutation of a glycine at position 2 in Matrix, which prevents the addition of a myristoyl group, abrogates this interaction as well as Gag ubiquitination and degradation. We also show that BCA2 promotes the lysosomal degradation of HIV-1 Gag. In agreement with this, and with the previously reported association of BCA2 with Rab7 –a small GTPase associated with lysosomal biogenesis [28]– cellular imaging analyses showed extensive co-localization of HIV-1 Gag with endo-lysosomal markers in cells expressing BCA2. The targeted depletion of endogenous BCA2 in tetherin-expressing and tetherin-deficient cells resulted in a significant increase in the release of virus particles, and therefore, in virus replication, suggesting that endogenous BCA2 has antiviral activity. Taken together, these observations indicate that BCA2 poses a significant barrier in the replication of HIV-1 and other retroviruses.

## Results

### BCA2 has tetherin-independent antiviral activity

**Figure 1:**
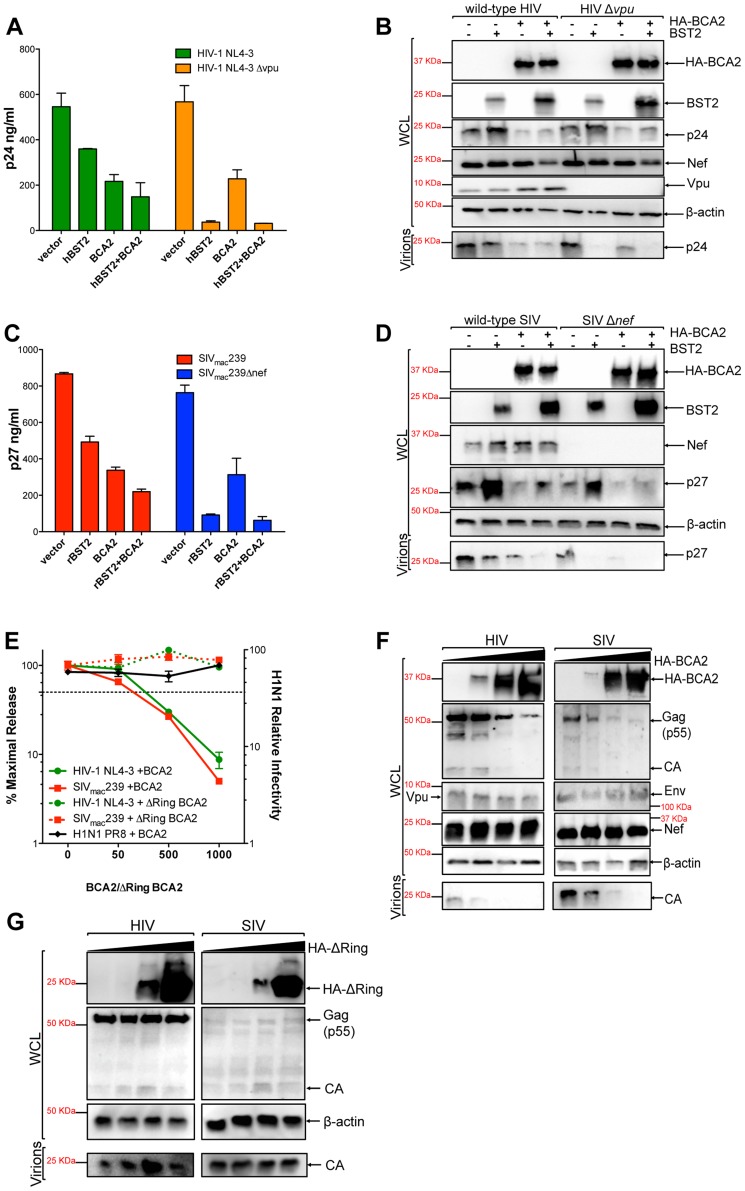
BCA2 has tetherin-independent antiviral activity. The antiviral activity of BCA2 was tested by co-transfecting 293T cells in duplicate with HIV-1 NL4-3, HIV-1 NL4-3 Δ*vpu*, SIV_mac_239 or SIV_mac_239 Δ*nef* proviral DNA in the presence of constructs coding for human or rhesus tetherin (hBST2, rBST2), HA-BCA2 or empty vector. Differences in DNA concentrations were offset by the addition of empty vector, and results were confirmed in three additional independent experiments. (A) Virus release for HIV-1 was measured by p24 antigen-capture ELISA 48 hours post-transfection. (B) The whole cell lysates (WCL) and virions generated from these transfections were analyzed by western blot to assess the expression levels of tetherin, HA-BCA2 and viral proteins (Nef, Vpu, Gag, CA: p24). (C) Virus release for SIV was assessed by p27 antigen-capture ELISA. (D) The expression levels of tetherin, HA-BCA2 and viral proteins were also assessed by western blot (Nef, Gag, CA: p27). (E) 293T cells were co-transfected with equal amounts of HIV-1 or SIV proviral DNA and increasing concentrations of expression vectors coding for HA-BCA2 or ΔRing BCA2. Differences in DNA concentration were offset by adding empty vector. Virus release was determined as described above, and expressed as the percentage of maximal virus release in the absence of HA-BCA2 or the ΔRing BCA2 mutant. To determine the infectivity of influenza H1N1, 293T cells were infected with 1 ml of H1N1 virions produced from HA-BCA2-expressing cells. Infectivity was determined 24 hours post-infection by measuring the amount of influenza hemagglutinin protein (HA) protein present on the cell surface. Similar to panels B and D, whole cell lysates and virions generated from transfections with HA-BCA2 (F) and ΔRing BCA2 (G) were analyzed by western blot. Error bars represent standard deviation of independent experiments.

To study the role of BCA2 in virus restriction, we examined the effects of this protein on virus release in the presence and absence of tetherin. For this, 293T cells, which do not express endogenous tetherin [15] (data not shown), were co-transfected with HIV-1 NL4-3, HIV-1 NL4-3 Δ*vpu*, SIV_mac_239 or SIV_mac_239 Δ*nef* proviral DNA, along with constructs expressing either human or rhesus macaque tetherin (hBST2 or rBST2), HA-tagged BCA2 or empty vectors (Figure 1). Transfections were performed in duplicate in 24-well plates, as described in the materials and methods section, and were repeated independently three additional times. The accumulation of virus particles in the cell culture supernatant was measured by HIV-1 p24 or SIV p27 antigen-capture ELISA 48 hours post-transfection, as previously described [22], [25], [29]. Since Vpu affords resistance to tetherin [15], [16], the restriction imposed by tetherin on virus release was significantly higher on *vpu*-deleted HIV-1 than on wild-type HIV-1. In agreement with previous observations [27], the antiviral activity of tetherin –particularly on wild-type HIV-1– was enhanced in cells expressing BCA2. Contrary to that report, a ∼2.5-fold reduction in virus release was detected from cells expressing BCA2, even in the absence of tetherin (Figure 1A). To confirm these observations, the cell lysates from these transfections, as well as the virions present in the culture supernatants, were analyzed by western blot (Figure 1B). Although we observed a reduction in the amount of virion-associated p24 from cells expressing tetherin, particularly for HIV-1 Δ*vpu* virions, a slight increase in the intracellular levels of p24 was detected, which likely reflects the presence of virions tethered to the cell surface. In cells expressing HA-BCA2, a significant decrease in the levels of virion-associated p24 was observed for both wild-type HIV-1 as well as for HIV-1 Δ*vpu*. This corresponded to a 4-fold reduction in the levels of intracellular p24. Nevertheless, BCA2 did not result in significant changes in the expression levels of other viral proteins, such as Nef or Vpu. Intermediate levels of cellular p24 were detected in cells co-expressing HA-BCA2 and tetherin, reflecting a defect in Gag expression or processing in the presence of HA-BCA2, and the restriction imposed by tetherin on trapping virions to the cell surface.

To determine if the effect of BCA2 on Gag expression and/or processing –and thereby on virus release– was specific for HIV-1, we performed similar assays with SIV_mac_239, a closely related lentivirus. Consistent with a role for Nef in tetherin antagonism [22], [23], the effects of tetherin on particle release were more pronounced in *nef*-deleted SIV than in wild-type virus (Figure 1C). Similar to HIV-1 NL4-3, expression of HA-BCA2 resulted in a ∼2.5-fold reduction in the amount of virus particles present in the culture supernatant (Figure 1C). These observations were also corroborated by western blot, where a decrease in cellular and virion-associated p27 was detected in cells expressing HA-BCA2 (Figure 1D), which is consistent with the data obtained for HIV-1 NL4-3. Therefore, BCA2 has tetherin-independent antiviral activity, which results in a defect in either Gag expression or processing, impairing virus assembly and release for both HIV-1 and SIV.

To better study the antiviral effects of BCA2, a titration curve was performed by co-transfecting 293T cells with increasing concentrations of a construct encoding HA-BCA2 and proviral DNA for wild-type HIV-1 NL4-3 or SIV_mac_239. Differences in DNA concentrations were offset by the addition of empty vector. Virus release was measured by HIV-1 p24 or SIV p27 antigen-capture ELISA and expressed as the percentage of maximal virus release in the absence of HA-BCA2. Consistent with the data presented in Figure 1A and 1C, increasing concentrations of the full-length BCA2 protein resulted in a dose-dependent reduction in virus release for both HIV-1 NL4-3 and SIV_mac_239 (Figure 1E; solid lines). Accordingly, a defect in cellular Gag expression was observed for both lentiviruses, but no defect in the expression levels of other viral proteins (Vpu, Nef or Env) was observed, indicating that the antiviral effects of BCA2 specifically target Gag (Figure 1F). Conversely, HA-BCA2 did not affect virus release nor infectivity for influenza virus H1N1 PR8, indicating that the antiviral activity of BCA2 may not be of broad range (Figure 1E, black line). To further characterize the mechanism of activity of BCA2, another titration curve was performed with a ligase-dead mutant of BCA2 (ΔRing BCA2). In this case, no reduction in virus release was observed for either HIV-1 NL4-3 or SIV_mac_239 (Figure 1E; dashed lines). In accordance with these results, no defect in Gag expression or processing was detected either (Figure 1G). Therefore, the antiviral effects of BCA2 require an intact RING-finger domain.

To rule out that the effects of BCA2 on Gag expression and virus production are not the result of artifacts due to protein overexpression, virus release assays for HIV-1 NL4-3 and SIV_mac_239 were performed in the presence of an irrelevant protein to the virus replication cycle (CD8-STOP). Since BCA2 is connected to the endo-lysosomal pathway through interactions with Rab7 [28], wild-type Dynamin 2 (Dyn2) was also included in these assays to test for proteins that may accelerate the rate of endocytosis. Consistent with the data shown in Figure 1, only cells expressing HA-BCA2 showed defects in virus release (Figure S1A-S1C). Additionally, we used a BCA2-specific antibody to compare the levels of ectopic HA-BCA2 relative to endogenous BCA2 in an additional titration curve for HIV-1 NL4-3. HA-BCA2 was expressed to a similar extent as endogenous BCA2 in transfections with 50 ng of the HA-BCA2 construct. At this concentration, there was already a noticeable effect on Gag expression and virus release (Figure S1D). Therefore, the decreased levels of Gag and virus production observed in cells expressing HA-BCA2 are not the result of artifacts due to protein overexpression.

The effects of BCA2 on virus release were also examined in an infectivity assay. 293T cells were co-transfected with HIV-1 NL4-3 or SIV_mac_239 proviral DNA in the presence and absence of HA-BCA2. Forty-eight hours post-transfection, virions accumulated in the culture supernatant were collected and used to infect the reporter GHOST X4/R5 cell line, which is stably transduced with a *tat*-inducible GFP reporter vector. The percentage of GFP^+^ cells was determined by FACS, and the infectivity of viruses produced from cells expressing HA-BCA2 relative to those produced from parental 293T cells was calculated (Figure S2A). These experiments were independently repeated in duplicate two additional times. Consistent with the virus release data, the amount of infectious particles produced from HA-BCA2-expressing cells was considerably reduced. The cell lysates and virions produced from these cells were also analyzed by western blot, confirming a substantial reduction in the levels of virion-associated capsid from cells expressing HA-BCA2 (Figure S2B). Therefore, BCA2 significantly decreases the offspring of infectious virions.

To explore if BCA2 exclusively targets lentiviral Gag proteins, we performed experiments with Mo-MLV, a *gammaretrovirus*. 293T cells were co-transfected with Mo-MLV proviral DNA along with constructs coding for HA-BCA2, the ligase-dead BCA2 mutant (ΔRing BCA2), tetherin as a positive control for restriction or empty vector. Transfections were performed in duplicate in three independent experiments, as explained above. Virus release for Mo-MLV was measured by MuLV p30 core antigen ELISA of the culture supernatant 48 hours post-transfection, and expressed as the percentage of maximal virus release in the absence of tetherin or HA-BCA2. In agreement with previous reports, tetherin imposed a significant barrier to the release of Mo-MLV virions [30]. Similar to HIV-1 and SIV, virus release was significantly impaired for Mo-MLV in cells expressing HA-BCA2, and no significant effects on virus release were observed in the presence of ΔRing BCA2 (Figure S3A). These results were confirmed by western blot, where a decrease in the expression levels of cellular Gag and virion-associated core protein p30 was detected in HA-BCA2-expressing cells (Figure S3B).

### Identification of residues in BCA2 required for antiviral activity

**Figure 2:**
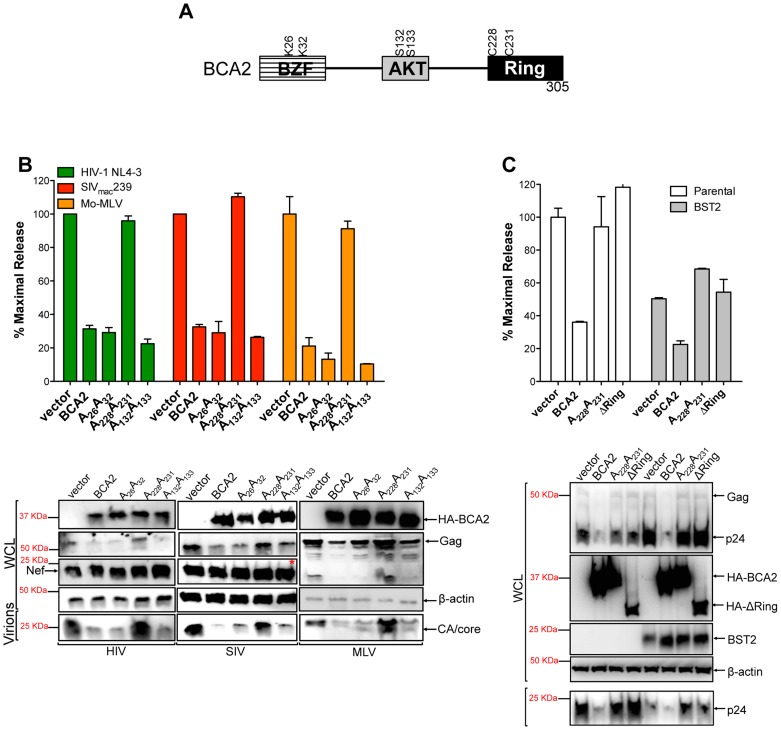
The E3 ligase activity of BCA2 is required for antiviral activity. (A) Schematic representation of BCA2 with the N-terminal BCA2 Zinc-finger domain (BZF), the AKT-phosphorylation sites, and the C-terminal RING-finger domain. Key residues are indicated. (B) Virus release assays for HIV-1 NL4-3, SIV_mac_239 and Mo-MLV were performed from cells expressing wild-type HA-BCA2 or the indicated BCA2 mutants, and expressed as the percentage of maximal release, as previously described. The whole cell lysates (WCL) and the virions produced were also analyzed by western blot to assess HA-BCA2, Gag, Nef, CA and β-actin expression. Red asterisk indicates MW of 37 KDa. (C) The tetherin-independent and tetherin-dependent antiviral activity of BCA2 was examined in a virus release assay from parental 293T and 293T cells stably expressing tetherin. Results were also corroborated by western blot of the whole cell lysates (WCL) and pelleted virions. Error bars represent standard deviation of independent experiments.

To define amino acids in BCA2 that contribute to antiviral activity, we introduced point mutations at important residues in BCA2, such as the E3 catalytic domain (C228C231), ubiquitination sites (K26K32) and phosphorylation sites (S132S133) [31] (Figure 2A). These mutants along with wild-type BCA2 were tested in three independent virus release assays for their antiviral activity against HIV-1 NL4-3, SIV_mac_239 and Mo-MLV, as previously described. Consistent with our initial observations, there was a ∼3-fold reduction in the amount of virus particles released into the culture supernatant of cells expressing wild-type HA-BCA2. Mutations in residues that are susceptible to become ubiquitinated as well as AKT-phosphorylated did not affect the antiviral activity of BCA2. However, mutations in the catalytic RING-finger domain resulted in the inability of BCA2 to inhibit virus release for these retroviruses (Figure 2B, upper panel). These observations were corroborated by western blot, where a decrease in cellular Gag and virion-associated capsid (CA) is only observed in the presence of BCA2, or functional BCA2 mutants (Figure 2B, bottom panel). Therefore, unlike the tetherin-dependent activity of BCA2, the E3 ligase activity is required for tetherin-independent restriction. To separate the tetherin-dependent (non-RING domain requiring) from the tetherin-independent (RING domain requiring) antiviral activity of BCA2, we performed additional assays in parental 293T cells and 293T cells stably expressing tetherin, where wild-type BCA2 and the ligase-dead mutants were tested for their antiviral activity. In contrast to Miyakawa's results [27], no enhancement in virus restriction was detected in the presence of tetherin and the RING-defective mutants, indicating that the reduction in virus release observed in the presence of HA-BCA2 and tetherin ­likely reflects a synergistic effect of the antiviral properties of both proteins (Figure 2C). Therefore, our results suggest that the antiviral activity of BCA2 is mainly tetherin-independent.

### BCA2 promotes the ubiquitination of HIV-1 Gag and other retroviral Gag proteins

**Figure 3:**
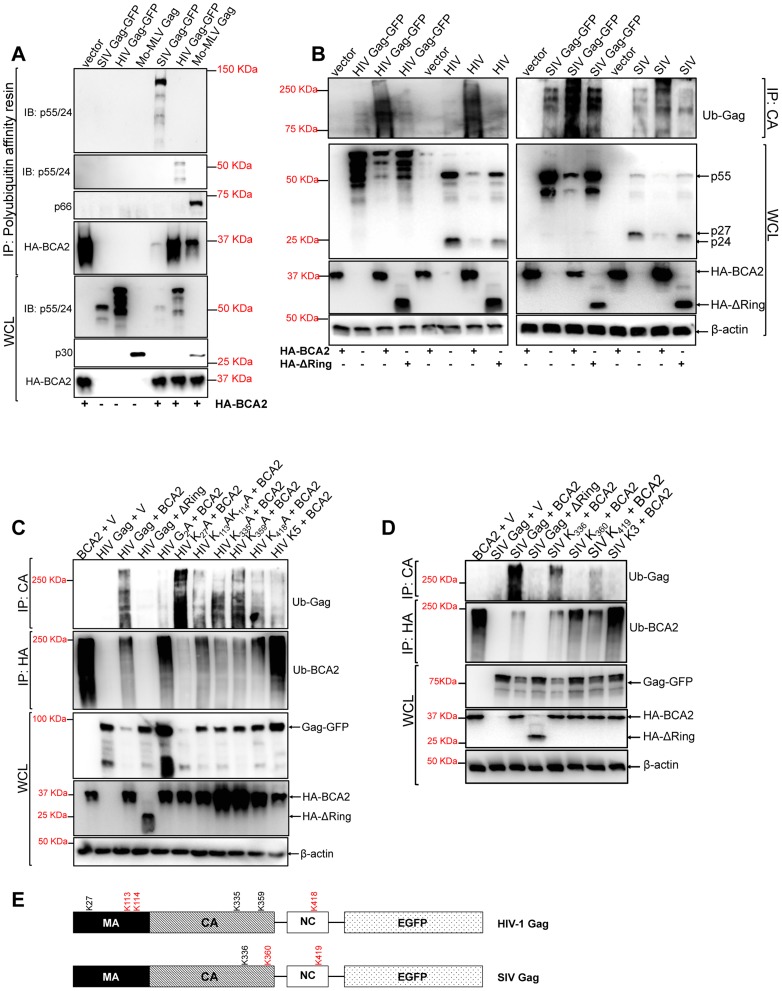
BCA2 induces the ubiquitination of retroviral Gag proteins. Retroviral Gag proteins were tested for BCA2-induced ubiquitination by co-transfecting 293T cells with vectors expressing codon-optimized HIV-1 NL4-3, SIV_mac_239 and Mo-MLV Gag proteins along with an expression vector coding for HA-BCA2 or empty vector. (A) Cell lysates were incubated with a polyubiquitin affinity resin and the bound fraction was eluted and analyzed by western blot. Membranes were probed with antibodies specific for p55/p24, MLV p30 and HA. Whole cell lysates were set aside for western blot analyses. (B) The BCA2-induced ubiquitination of Gag-GFP as well as native Gag was analyzed in side-by-side experiments. 293T cells were co-transfected in duplicate with each Gag construct, HA-BCA2, ΔRing BCA2 or empty vector. Cell lysates were set aside for regular western blotting, and the rest of the lysates were immunoprecipitated with an antibody anti-CA. Membranes were developed with an anti-Ubiquitin (Ub) antibody. (C) The ubiquitination of HIV-1 Gag, or HIV-1 Gag mutants, in cells expressing HA-BCA2 was also analyzed by immunoprecipitation. Cell lysates were set aside for western blot analyses and the rest of the samples were immunoprecipitated with an anti-CA specific antibody. Membranes were probed with an anti-Ubiquitin antibody. Similarly, the ubiquitination levels of HA-BCA2 were also determined. In this case, samples were immunoprecipitated using an anti-HA antibody. Results were confirmed in two additional independent assays. (D) A similar approach was used to analyze the ubiquitination levels of SIV Gag, or SIV Gag mutants, in the presence and absence of HA-BCA2. (E) Schematic representation of the putative lysine residues susceptible to become ubiquitinated by BCA2 in HIV-1 and SIV Gag (red). IP: immunoprecipitation. IB: immunoblot. WCL: whole cell lysate. V: empty vector.

Since the E3 ligase activity of BCA2 is required for antiviral activity, we hypothesize that the mechanism by which BCA2 impairs the expression of Gag requires Gag ubiquitination. To test this, we performed polyubiquitination assays. 293T cells were co-transfected with HIV-1, SIV and Mo-MLV Gag constructs in the presence and absence of HA-BCA2. Cell lysates were incubated with a polyubiquitin affinity resin (Thermo Scientific). After several washing steps, the bound fraction was eluted and analyzed by western blot. Retroviral Gag proteins were found to bind the polyubiquitin affinity resin only in the presence of HA-BCA2, suggesting that BCA2 promotes their ubiquitination (Figure 3A). As a control, HA-BCA2 was also detected in the eluted fraction, since BCA2 has auto-ubiquitination activity [32]. However, its ubiquitination levels were variable in these samples, although the input levels of HA-BCA2 were similar across the assay (Figure 3A). This may reflect a previously reported characteristic of the E3 ligase activity of BCA2 in substrate ubiquitination versus auto-ubiquitination, where the auto-ubiquitination activity of BCA2 increases in the absence of target proteins susceptible to become ubiquitinated [33]. This is consistent with the fact that the lower levels of ubiquitinated HA-BCA2 are found in the presence of the Gag protein showing more binding to the polyubiquitin affinity resin (SIV Gag). However, more ubiquitinated HA-BCA2 is found in the presence of HIV-1 Gag, which bound less efficiently to the resin. These observations were corroborated in two additional independent experiments.

Although these results indicated that Gag proteins become ubiquitinated in the presence of BCA2, their electrophoretic pattern did not match with the profile of ubiquitinated proteins, which normally display a characteristic smear. Since BCA2 has auto-ubiquitination activity, the presence of Gag proteins in the pulled-down fraction may be due to indirect effects on Gag-BCA2 association. To further investigate if Gag undergoes ubiquitination by BCA2, we performed immunoprecipitation assays in which retroviral Gag proteins were pulled-down in the presence and absence of HA-BCA2, and the presence of Ubiquitin chains in Gag was determined by probing western blot membranes with an anti-Ubiquitin (Ub) antibody. To control for the presence of the GFP tag associated to Gag, as a potential target for BCA2-ubiquitination, we performed ubiquitination assays in parallel for Gag-GFP and native Gag expressed from proviral DNA. The levels of ubiquitination in HIV-1 and SIV Gag increased in cells expressing HA-BCA2, either for native Gag as for Gag-GFP. However, the levels of Ub-Gag were similar to those observed in the presence of the empty vector control in cells expressing the ligase-dead mutant of BCA2 (ΔRing BCA2) (Figure 3B). Hence, these results support that BCA2 promotes Gag ubiquitination.

We next ran computational analyses to identify lysine residues in HIV-1 and SIV Gag susceptible to become ubiquitinated (Tables 1 and 2). Alanine substitutions were introduced at those positions, and the ubiquitinated status of those mutants was tested along with wild-type Gag and a Gag-myristoyl mutant (G_2_A) (Figure 3C). HIV-1 Gag was found to become ubiquitinated in the presence of HA-BCA2, but its ubiquitination levels were almost undetectable in cells co-transfected with the empty vector control. Consistent with the requirement of an intact RING-finger domain for the E3 ligase activity, almost no Ub-Gag was detected in cells expressing ΔRing BCA2, and auto-ubiquitination was also lost for this mutant. Interestingly, prevention of the addition of a myristoyl group in Matrix resulted in a significant loss of Gag ubiquitination, indicating that Gag must be associated to cellular membranes in order to be targeted by BCA2. Mutation of K27 in HIV-1 Gag did not affect BCA2-mediated ubiquitination. However, alanine substitutions at positions 113, 114, and 418 in HIV-1 Gag significantly reduced the levels of Gag-associated Ubiquitin, and combination of all alanine substitutions (K5) further decreased the levels of Ub-Gag (Figure 3C). In accordance with the previously mentioned enzymatic characteristics of BCA2, the auto-ubiquitination levels of HA-BCA2 were considerably lower in the presence of HIV-1 Gag proteins susceptible to become ubiquitinated than in the presence of HIV-1 Gag mutants that were resistant to ubiquitination. Remarkably, the defect in HIV-1 Gag expression was only observed when the levels of ubiquitinated Gag increased (Figure 3B and 3C).

**Table 1:** Lysine residues in HIV-1 Gag predicted to become ubiquitinated.

Peptide	Position	Score	Threshold
RLPGGK**K**KYLKHI	27	2.55	0.3
EEQNKSK**K**KAQQAAA	113	1.85	0.3
EQNKSKK**K**AQQAAAD	114	2.34	0.3
PDCKTIL**K**ALGPAAT	335	2.02	0.3
GVGGPGH**K**ARVLAEA	359	2.91	0.3
KGCWKCG**K**EGHQMKD	418	1.74	0.3

The complete coding sequence of HIV-1 Gag was analyzed by a Bayesian Discriminant Method algorithm (http://bdmpub.biocuckoo.org/). The selected amino acids obtained the highest score for the prediction.

**Table 2:** Lysine residues in SIV Gag predicted to become ubiquitinated.

Peptide	Position	Score	Threshold
PDCKLVL**K**GLGVNPT	336	2.51	0.3
GVGGPGQ**K**ARLMAEA	360	2.13	0.3
QGCWKCG**K**MDHVMAK	419	1.78	0.3

The full coding sequence of SIV Gag was analyzed by a Bayesian Discriminant Method algorithm (http://bdmpub.biocuckoo.org/). The selected amino acids obtained the highest score for this prediction.

Similar results were obtained for SIV Gag (Figure 3D). The ubiquitination levels of SIV Gag increased in the presence of HA-BCA2, and almost no Ub-Gag was detected in the presence of the ΔRing BCA2 mutant. Mutation of K336 in SIV Gag had practically no effect on its Ubiquitin levels, and a significant reduction in Ub-Gag was observed with the Gag mutant containing an alanine substitution at position 360 and also, although less dramatic, with the alanine mutant at position 419. Combination of these mutations (K3) further reduced Ub-Gag (Figure 3D), indicating that BCA2 primarily targets K360 and also K419 in SIV Gag for ubiquitination (a schematic representation of the positions of these lysine residues is provided in Figure 3E). Consistent with the decreased auto-ubiquitination levels of HA-BCA2 observed in the presence of HIV-1 Gag, HA-BCA2 was more ubiquitinated in the absence of SIV Gag, or in the presence of the SIV Gag K3 mutant, than in the presence of SIV Gag proteins susceptible to become ubiquitinated (Figure 3D). Accordingly, the cellular levels of SIV Gag were substantially restored for Gag mutants resistant to BCA2-mediated ubiquitination (Figure 3D). Therefore, the antiviral effects of BCA2 on HIV-1 and SIV Gag expression correlate with the ability of BCA2 to promote the ubiquitination of these Gag proteins.

### BCA2 physically interacts with Gag

**Figure 4:**
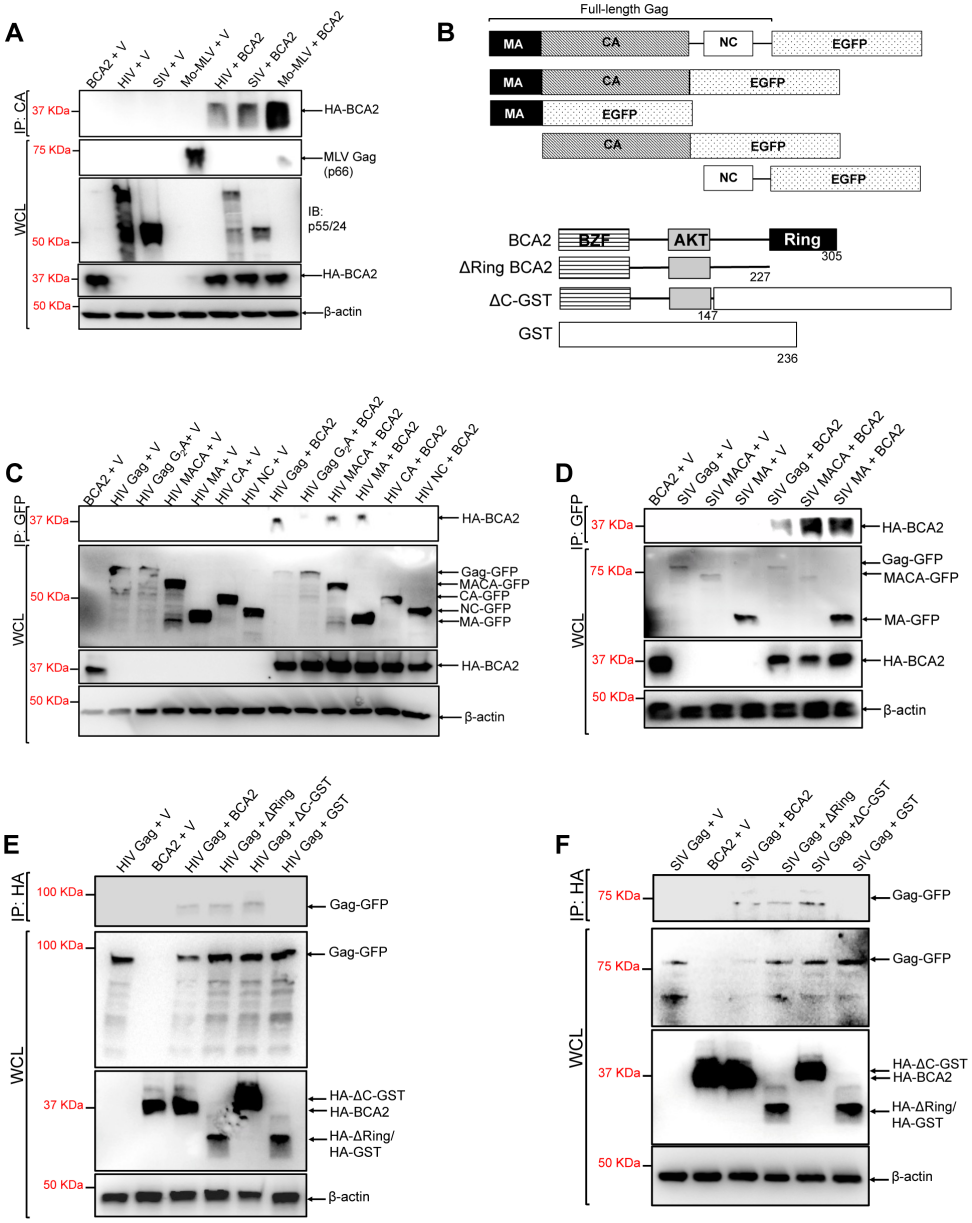
The N-terminus of BCA2 interacts with the Matrix region of Gag. (A) To investigate if retroviral Gag proteins interact with BCA2, 293T cells were co-transfected with HIV-1, SIV and Mo-MLV Gag constructs and either empty vector or a vector encoding HA-BCA2. Cell lysates were immunoprecipitated with a CA-specific antibody and membranes were probed with an anti-HA antibody. Results obtained from these assays were corroborated independently twice. (B) Schematic representation of the Gag and HA-BCA2 deleted constructs used. Similar to panel A, HIV (C) and SIV (D) Gag deleted mutants were tested for an interaction with HA-BCA2 by co-immunoprecipitation. Likewise, HA-BCA2 deleted mutants were tested for an interaction with HIV (E) and SIV (F) Gag proteins by co-immunoprecipitation. In this case, lysates were immunoprecipitated with an anti-HA antibody and western blot membranes were probed with a GFP-specific antibody. Whole cell lysates (WCL) were set aside to check the input levels of these proteins and β-actin. IP: immunoprecipitation. V: empty vector.

The BCA2-induced ubiquitination of HIV-1 and SIV Gag suggests that BCA2 physically interacts with Gag proteins to promote their ubiquitination. To explore this, retroviral Gag proteins were tested for a physical interaction with BCA2 by co-immunoprecipitation assays. HIV-1, SIV and Mo-MLV Gag were immunoprecipitated from lysates of 293T cells co-transfected with constructs expressing HA-BCA2 and each of these retroviral Gag proteins (Figure 4A). Immunoprecipitated proteins were separated by SDS-PAGE, and western blot membranes were probed with a monoclonal antibody to HA. HA-BCA2 was strongly pulled down in the presence of Mo-MLV Gag, whereas a weaker interaction was detected with SIV and HIV-1 Gag (Figure 4A). These results were confirmed two additional times. To identify the region in Gag that interacts with BCA2, new co-immunoprecipitation assays were performed with Gag-GFP deleted mutants (Figure 4B, upper diagrams). HA-BCA2 was pulled down in the presence of the full-length HIV-1 Gag-GFP protein as well as MACA-GFP and MA-GFP, but not in the presence of CA-GFP and NC-GFP, indicating that BCA2 and HIV-1 Gag interact through the Matrix region of Gag. These results were confirmed by performing reciprocal co-immunoprecipitation assays, where Gag-GFP constructs containing the Matrix region of HIV-1 Gag were immunoprecipitated with HA-BCA2 (Figure S4). In agreement with our ubiquitination data, this interaction was significantly reduced in cells expressing the HIV-1 Gag-G_2_A-GFP mutant, further confirming that Gag must be associated to cellular membranes to be targeted by BCA2 (Figure 4C). Similar results were obtained for SIV Gag. HA-BCA2 was pulled down in the presence of the full-length SIV Gag-GFP protein or constructs containing the Matrix region of Gag (Figure 4D).

To identify the region in BCA2 interacting with HIV-1 and SIV Gag, similar experiments were performed in the presence of HA-BCA2 deleted mutants (Figure 4B, lower diagrams). In this case, HIV-1 and SIV Gag both immunoprecipitated with the full-length HA-BCA2 protein as well as with ΔRing BCA2 and a GST-BCA2 fusion protein lacking the C-terminus of BCA2 (residues 148–305: ΔC-GST), but not with GST, indicating that the interaction between Gag and BCA2 takes place within the first half of the protein (residues 1–147) (Figure 4E and 4F). These results were confirmed in two independent assays. In order to perform a loss of binding assay, we engineered a BCA2 deleted mutant lacking the N-terminal Zinc-finger domain. However, the expression levels of this construct were practically undetectable, even when fused to GST (data not shown). Thus, we decided not to include this mutant in our mapping studies.

### BCA2 promotes the lysosomal degradation of HIV-1 and SIV Gag

**Figure 5:**
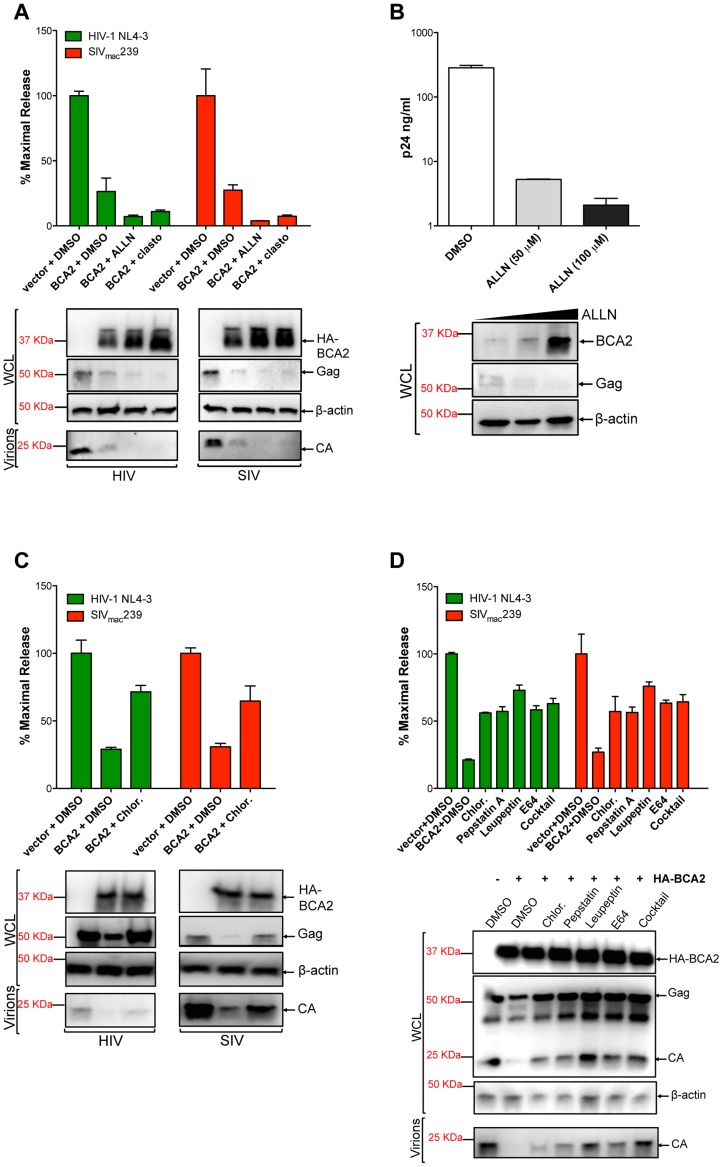
BCA2 promotes the lysosomal degradation of HIV-1 and SIV Gag. To determine if BCA2 promotes the proteasomal or lysosomal degradation of retroviral Gag proteins, virus release assays for HIV-1 and SIV were performed in the presence of proteasomal inhibitors (A-B) and lysosomal inhibitors (C-D). Virus release was measured by HIV-1 p24 or SIV p27 antigen-capture ELISA and expressed as the percentage of maximal virus release in the absence of HA-BCA2. The cell lysates (WCL) and virions derived from these transfections were analyzed by western blot. Clasto: clasto-Lactacystin β-lactone. Chlor: chloroquine. Error bars represent standard deviation of independent experiments.

The fact that the BCA2-induced defect in Gag expression correlates with Gag ubiquitination, suggests that BCA2 promotes the degradation of retroviral Gag proteins. To explore this, the antiviral effects of BCA2 were analyzed in the presence of proteasomal and lysosomal inhibitors. 293T cells were co-transfected with HIV-1 NL4-3 or SIV_mac_239 proviral DNA and a construct coding for HA-BCA2 or empty vector. Twenty-four hours post-transfection, the cells were washed and fresh medium was added containing either DMSO, proteasomal inhibitor drugs (ALLN, clasto-Lactacystin β-lactone) or drugs that impair lysosomal function (Chloroquine, Pepstatin A, Leupeptin and/or E64). The accumulation of virus particles in the culture supernatant was measured by HIV-1 p24 or SIV p27 antigen-capture ELISA 48 hours post-transfection, and expressed as the percentage of maximal release in the absence of HA-BCA2 (Figure 5). The results obtained were confirmed in four independent experiments. As expected, transfections with HA-BCA2 constructs resulted in a ∼3-fold reduction in the amount of virus particles present in the culture supernatant compared to transfections with an empty vector. The addition of proteasomal inhibitors to cells expressing HA-BCA2 further decreased virus release for both, HIV-1 NL4-3 and SIV_mac_239. Western blot analyses of the cell lysates and pelleted virions confirmed these observations, and revealed a substantial increase in the steady-state levels of HA-BCA2 in the presence of proteasomal inhibitors (Figure 5A). This is consistent with a previous report demonstrating that the auto-ubiquitination activity of BCA2 reduces its stability, so it is rapidly degraded through the proteasome. Therefore, in the presence of drugs that block proteasomal degradation, the turnover of BCA2 is reduced [34]. In fact, treatment of parental 293T cells with increasing concentrations of ALLN led to increased levels of endogenous BCA2 and resulted in a dose-dependent decrease in cellular levels of HIV-1 Gag and virus release (Figure 5B). The effects of ALLN on Gag expression and virus release were reversed in cells depleted from BCA2 (Figure S5A and S5B), further supporting that this effect was BCA2-specific. Therefore, the higher restriction in virus release observed in the presence of these proteasomal inhibitors reflects an increase in BCA2 steady-state levels, and thereby in antiviral activity, and indicates that the BCA2-induced reduction of Gag does not involve proteasomal degradation.

We next examined if BCA2 induces the lysosomal degradation of Gag by adding Chloroquine, a lysosomotropic drug, to the culture medium. The antiviral effects of BCA2 on virus release were impaired in the presence of this drug, since there was >2-fold rescue in particle release (Figure 5C). This was confirmed by western blot. In this case, no increase in the cellular levels of HA-BCA2 was observed, and the levels of cellular Gag and virion-associated capsid were significantly restored (Figure 5C). To confirm these results, additional assays were performed in the presence of drugs that specifically inhibit lysosomal proteases. The addition of Pepstatin A (inhibitor of aspartic proteases) alone or in combination with Leupeptin (serine and cysteine proteases inhibitor) and E64 (cysteine protease inhibitor) to cells expressing HA-BCA2 also resulted in a ∼2-fold increase in virus release compared to the amount of virus particles released from cells expressing HA-BCA2 and treated with DMSO (Figure 5D). These observations were corroborated by western blot analyses, showing a substantial rescue in Gag expression in cells treated with lysosomal inhibitors, which also results in a rescue in the levels of virion-associated capsid (Figure 5D, bottom panel). To test if these drugs would intrinsically increase virus release, similar assays were performed side-by-side in vector-transfected and HA-BCA2-expressing cells. The addition of inhibitors of lysosomal proteases only resulted in increased virus release under conditions of ectopic expression of BCA2 (Figure S5C). The fact that the inhibitory effects of BCA2 on virus release were not fully reverted in the presence of these drugs may suggest that additional routes or factors contribute to the antiviral activity of BCA2, but may also reflect an additional role for components of the endo-lysosomal/autophagy pathway in virus assembly and release [35], [36], [37], [38], [39]. To further explore the role of the endo-lysosomal pathway in the mechanism of BCA2 restriction, we examined the effects of a dominant-negative mutant of Rab7A (Rab7 N125I) –a BCA2-interacting GTPase associated with both the endosome and lysosome– on the activity of endogenous and ectopically expressed BCA2 (Figure S5D). Expression of Rab7 N125I increased virus release from parental 293T cells (solid bars) as well as from HA-BCA2 expressing cells (bars with a pattern), and this increase in virus release correlates with a substantial rescue in Gag and CA levels (Figure S5D, bottom panel). Therefore, these results support that BCA2 induces the lysosomal degradation of retroviral Gag proteins, which is in agreement with the fact that BCA2 interacts with Rab7 [27], [28].

### The targeted depletion of BCA2 results in increased virus release and replication, even in cells not expressing tetherin

**Figure 6:**
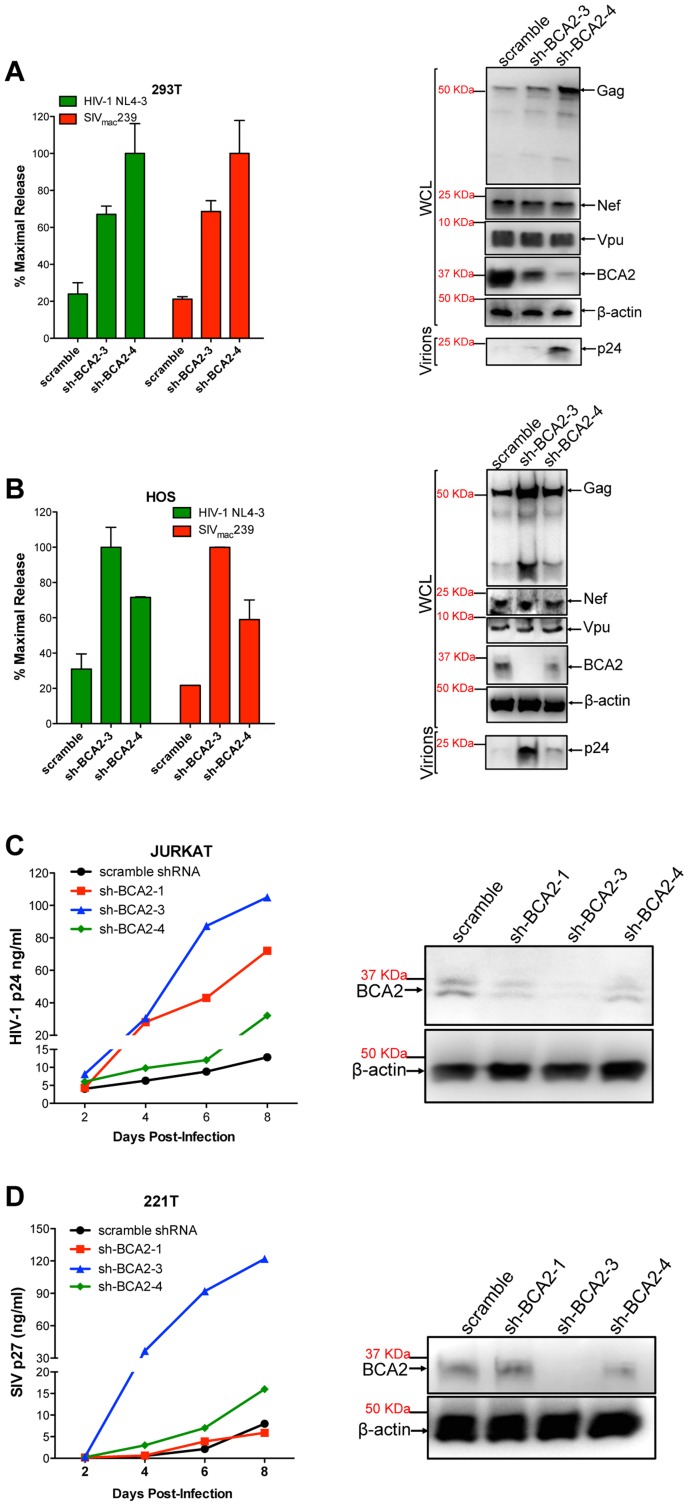
The targeted depletion of endogenous BCA2 results in increased virus release and replication. Endogenous BCA2 was depleted by shRNA from 293T cells (A) and HOS cells (B) by transient transfection. Cells were subsequently transfected with HIV-1 NL4-3 or SIV_mac_239 proviral DNA, and virus release was measured 48 hours later. Depletion of endogenous BCA2 was also achieved in CD4^+^ T cells by transduction. Next, virus replication was assessed for HIV-1 in Jurkat cells (C) and for SIV in 221 T cells (D) by HIV-1 p24 and SIV p27 antigen-capture ELISA, respectively, at selected time points. The depletion of endogenous BCA2 was confirmed by western blot for each of these cell lines, by comparing the levels of endogenous BCA2 in the whole cell lysate (WCL) to those of β-actin. Error bars represent standard deviation of independent experiments.

Several cell types express basal levels of BCA2, including 293T cells. Therefore, we wanted to investigate if endogenous BCA2 has antiviral activity against HIV-1 and SIV. 293T cells and HOS cells, which do not express endogenous tetherin [15], were transfected with lentiviral-based vectors coding for BCA2-specific shRNAs (sh-BCA2-1, sh-BCA2-3 and sh-BCA2-4) or scrambled shRNA. After two rounds of shRNA transfections, the cells were transfected with HIV-1 NL4-3 or SIV_mac_239 proviral DNA. Virus release was measured 48 hours post-transfection as previously described, and expressed as the percentage of maximal release of three independent assays. The knock down of BCA2 was confirmed by western blot using a BCA2-specific antibody, and comparing the expression of endogenous BCA2 to β-actin. The targeted depletion of BCA2 in 293T cells resulted in a 3 to 5-fold increase in virus release compared to scrambled shRNA-treated cells (Figure 6A). This was corroborated by western blot analyses showing an increase in Gag expression in cells depleted from BCA2, without affecting the expression levels of other viral proteins. Similar results were obtained in HOS cells, where the best knockdown of BCA2 afforded ∼4-fold increase in virus release (Figure 6B).

To investigate the relevance of endogenous BCA2 for HIV-1 and SIV replication in target cells, human Jurkat CD4^+^ T cells and 221T cells, a *Herpesvirus saimiri*-immortilized rhesus macaque CD4^+^ T cell line [40], were transduced with scrambled shRNA or BCA2-specific shRNAs. The knockdown of BCA2 was assessed by western blot, as described above. Virus replication for HIV-1 NL4-3 increased by ∼10-fold at day 8 post-infection in cells transduced with sh-BCA2-3, which afforded the best effect on depleting BCA2 in Jurkat cells. In cells transduced with sh-BCA2-4, which only had a minor reduction in the endogenous levels of BCA2, there was only a 2.5-fold increase in virus replication. An intermediate phenotype (∼5-fold enhancement) was observed in cells transduced with sh-BCA2-1, which afforded intermediate effects on the knockdown of BCA2 (Figure 6C). Similar results were obtained for SIV in 221T cells, where virus replication was 15-fold higher in cells treated with sh-BCA2-3, which afforded the best knockdown of endogenous BCA2, compared to cells treated with scrambled shRNA (Figure 6D). Therefore, these observations indicate that endogenous BCA2 has antiviral activity.

### BCA2 induces changes in the subcellular distribution of Gag

**Figure 7:**
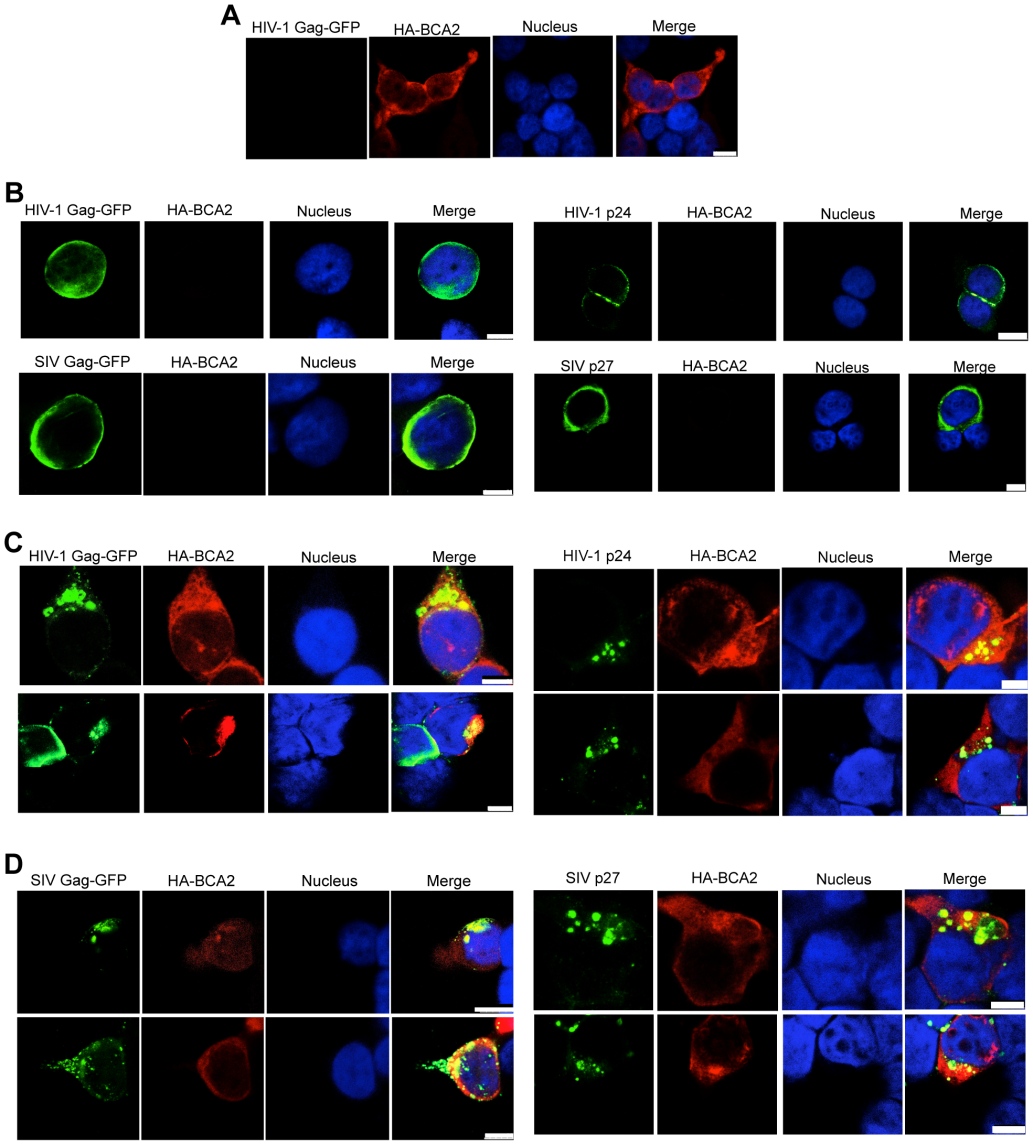
BCA2 leads to the accumulation of Gag proteins within intracellular compartments. The effects of BCA2 on Gag distribution were investigated by confocal microscopy. 293T cells were co-transfected with constructs coding for HA-BCA2 and HIV-1 Gag-GFP, SIV Gag-GFP or empty vector. Similar assays were performed in the presence of HIV-1 NL4-3 and SIV_mac_239 proviral DNA. Cells were stained for HA-BCA2 (red), Gag-GFP or Capsid (p24 or p27) (green) and the nuclei (blue). (A) Cellular distribution of HA-BCA2 in Gag-deficient cells. (B) Cellular distribution of HIV-1 Gag-GFP, SIV Gag-GFP (left panels) and HIV-1 p24 or SIV p27 (right panels) in HA-BCA2-deficient cells. (C) Cellular distribution of HIV-1 Gag-GFP and HIV-1 p24 in cells expressing HA-BCA2. (D) Cellular distribution of SIV Gag-GFP and SIV p27 in HA-BCA2-expressing cells. The white scale bar corresponds to 10 µm for images with more than one cell, and 7.5 µm for images with single cells.

Changes in the subcellular distribution of HIV-1 and SIV Gag were examined in HA-BCA2-expressing and parental cells by confocal microscopy. 293T cells were transfected with constructs coding for either HIV-1 Gag-GFP, SIV Gag-GFP, or proviral DNA for HIV-1 and SIV, along with a vector encoding HA-BCA2 or empty vector. Cells were stained for GFP or CA (green), HA-BCA2 (red) and the nuclei (blue). In HA-BCA2 expressing cells, BCA2 was found to localize primarily in the cytoplasm, but also in the nucleus, as previously reported [32] (Figure 7A). In parental cells, native HIV-1 Gag, HIV-1 Gag-GFP, native SIV Gag and SIV Gag-GFP localized mainly at the plasma membrane and also at membrane-proximal locations (Figure 7B). In cells expressing both, HA-BCA2 and Gag-GFP, Gag was found within the cytoplasm, at punctuated locations (Figure 7C and 7D, left panels). The effect of HA-BCA2 on the redistribution of Gag was also observed in native Gag (Figure 7C and 7D, right panels).

**Figure 8:**
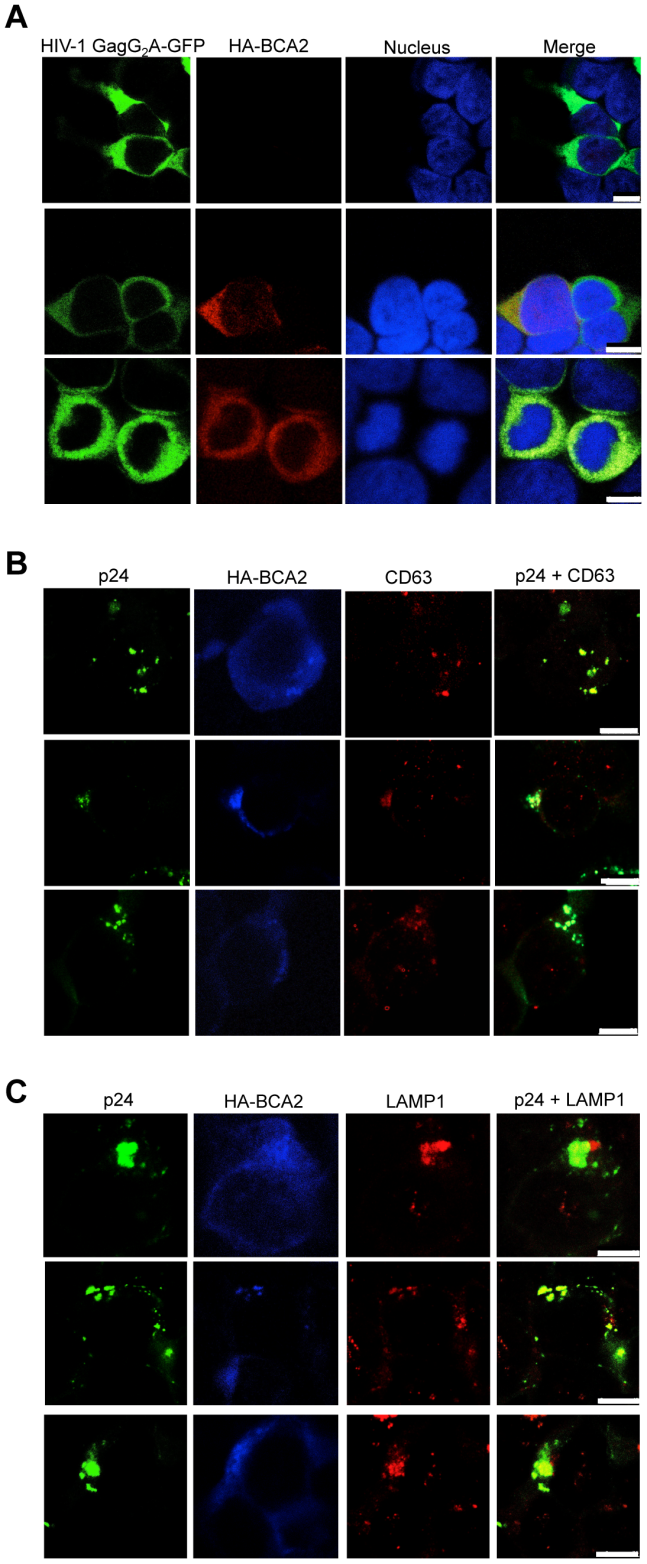
BCA2 re-distributes HIV-1 Gag, but not HIV-1 Gag G_2_A, to endo-lysosomal compartments. (A) Distribution of the HIV-1 Gag-G_2_A-GFP mutant in HA-BCA2^−^ (top panel) and HA-BCA2^+^ (bottom panels) cells. (B-C) The intracellular localization of native HIV-1 Gag in the presence of HA-BCA2 was determined by staining cells for p24 (green), HA-BCA2 (blue) and either CD63, or LAMP1 (red). The white scale bar corresponds to 10 µm for images with more than one cell, and 7.5 µm for images with single cells.

Consistent with our data showing that myristoylation of Matrix is required for BCA2 antiviral activity, the BCA2-induced changes in the redistribution of Gag disappeared in cells expressing the HIV-1 Gag-G_2_A-GFP mutant, since HIV-1 Gag-G_2_A-GFP was found evenly distributed in the cytoplasm (Figure 8A, bottom panels), displaying the same localization as in parental cells (Figure 8A, top panels). To reveal the intracellular localization of native Gag in HA-BCA2-expressing cells, 293T cells were stained for HIV-1 p24 (green), HA-BCA2 (blue) and specific intracellular markers (red), and the localization of Gag relative to these cellular markers was analyzed. Consistent with a role for BCA2 in routing Gag for lysosomal degradation, Gag co-localized with endo-lysosomal markers (CD63 and LAMP1) (Figure 8B and 8C), but not with markers of the *trans*-Golgi network (Figure S6). Similar results were obtained for SIV Gag (data not shown). Therefore, BCA2 induces drastic changes in the subcellular localization of HIV-1 and SIV Gag, which are primarily found at punctuated locations in the cytoplasm, co-localizing with endo-lysosomal markers.

## Discussion

Mammalian cells express a number of proteins that block HIV replication. These include APOBEC3 proteins, TRIM5 members, Mx2/MxB, SAMHD1 and tetherin/BST2 [5], [6], [8], [9], [10], [11], [14], [15], [16], [17], [18], [19], [41]. Tetherin is an interferon-inducible transmembrane protein that traps nascent virions to the plasma membrane, impairing virus release and spread. A recent study identified BCA2, a RING-finger E3 ubiquitin ligase, as a co-factor in the restriction imposed by tetherin on HIV-1 [27]. BCA2 was shown to interact with sequences in the cytoplasmic domain of tetherin to promote the internalization and lysosomal degradation of tethered virions. However, its E3 ligase activity was dispensable for virus restriction. Ectopic expression of BCA2 in tetherin-deficient cells (HOS) showed no effects on HIV release. Therefore, it was concluded that BCA2 lacks antiviral activity in the absence of tetherin. Here we show for the first time that BCA2 possesses tetherin-independent antiviral activity that leads to the ubiquitination and lysosomal degradation of HIV-1 and other retroviral Gag proteins, thereby, impairing virus assembly and release.

Increasing concentrations of BCA2 in 293T cells, which do not express endogenous tetherin [15], resulted in a dose-dependent decrease in the amount of HIV-1 virus particles released into the cell culture supernatant, suggesting that BCA2 has antiviral activity even in the absence of tetherin. Western blot analyses revealed that this defect in virus release corresponds to a reduction in the expression levels of Gag, with no effects on other viral proteins. Therefore, the antiviral activity of BCA2 seems to specifically target the Gag polyprotein. Similar results were obtained for SIV and Mo-MLV, but no antiviral activity was observed against influenza virus H1N1 PR8, indicating that BCA2 possesses antiviral activity against several retroviruses, but its inhibitory effects are not of broad range.

The discrepancy of these results with those from Miyakawa et al. [27] may be due to the use of different cell lines (293T instead of HOS cells). To test this, we performed additional assays in HOS cells. However, we had similar results to those obtained in 293T cells (data not shown). This is consistent with our shRNAs assays, which demonstrate that endogenous BCA2 in HOS cells has antiviral activity. Since there are many commercially available HOS cells, we hypothesize that the ones used by Miyakawa might be deficient in factors required for BCA2 antiviral activity. Nevertheless, unlike Miyakawa's observations, our data clearly shows that, besides a synergistic effect on virus restriction in the presence of tetherin, BCA2 mainly functions as a tetherin-independent antiviral factor that impairs virus release.

Unlike the tetherin-dependent antiviral activity of BCA2 [27], the E3 ligase activity is required for tetherin-independent restriction, since overexpression of BCA2 mutants with impaired ligase activity did not interfere with virus release or Gag expression. Consistent with these results, ubiquitination assays revealed that HIV-1 and SIV Gag become ubiquitinated in the presence of BCA2. In accordance with a previously reported intrinsic preference for substrate ubiquitination versus auto-ubiquitination [33], the auto-ubiquitination activity of BCA2 was dramatically reduced in the presence of Gag proteins susceptible to BCA2-mediated ubiquitination. The introduction of alanine substitutions at lysine residues in Gag predicted to become ubiquitinated (particularly at K113, K114, and K418 in HIV-1 Gag; K360 and K419 in SIV Gag) impaired the BCA2-induced ubiquitination of Gag, a fact that was more evident when those alanine substitutions were combined. The fact that BCA2 promotes the ubiquitination of retroviral Gag proteins raised the possibility that BCA2 and Gag physically interact. Co-immunoprecipitation assays revealed a physical interaction between these two proteins, and we further mapped this association to the Matrix region in Gag and the first half portion of BCA2.

Our ubiquitination assays also showed that the BCA2-induced ubiquitination of Gag directly correlates with a reduction in Gag levels, suggesting that BCA2 may promote the ubiquitination and degradation of retroviral Gag proteins. Assays with proteasomal inhibitors showed further inhibition in virus release in cells expressing BCA2. Western blot analyses revealed a substantial increase in the steady-state levels of BCA2 in the presence of these drugs, which is in agreement with previous reports [34]. Indeed, treatment of parental 293T cells with proteasomal inhibitors led to increased BCA2 levels and a corresponding reduction in Gag and virus release. Therefore, not only this assay proves the existence of BCA2-specific antiviral activity, but also that the BCA2-dependent defect in Gag levels does not require the proteasome. On the other hand, assays with lysosomal inhibitors showed a significant rescue in virus release in cells expressing BCA2. However, virus release was not restored to the levels achieved in cells transfected with the empty vector control, suggesting that BCA2 promotes, at least in part, the lysosomal degradation of Gag, but other pathways may also be involved in the mechanism of restriction. The BCA2-induced lysosomal degradation of Gag is consistent with a previously reported interaction between BCA2 and Rab7 [27], [28], a GTPase associated with lysosomal biogenesis. Indeed, assays with a dominant-negative mutant of Rab7 impaired BCA2 function and led to increased Gag levels, and therefore, a substantial rescue in virus release. Moreover, BCA2 has recently been shown to route EGFR for endosomal sorting [42], [43], further corroborating the connection of BCA2 with the endo-lysosomal pathway.

BCA2 localizes uniformly in the cytoplasm and nucleus [32], whereas HIV-1 Gag is primarily found at the plasma membrane or at membrane-proximal locations [44], [45]. The effects of BCA2 on the subcellular distribution of HIV-1 and SIV Gag were studied by confocal microscopy. Whereas HIV-1 and SIV Gag localized mainly at the plasma membrane in parental 293T cells, the intracellular distribution of these Gag proteins was reorganized in cells expressing HA-BCA2, since Gag was found at punctuated locations in the cytoplasm. Strikingly, mutation of the Matrix myristoylation site, which prevented the localization of Gag at the plasma membrane [45], also prevented the intracellular re-distribution of Gag observed in cells expressing HA-BCA2. These results are consistent with our data showing that Gag must be associated to the plasma membrane in order to be susceptible to BCA2. An analysis of the subcellular distribution of Gag in HA-BCA2-expressing cells revealed that Gag co-localized with endo-lysosomal markers, further supporting a role for BCA2 in targeting Gag for lysosomal degradation.

Although BCA2 was initially found to be overexpressed in >50% of invasive breast cancers [32], it is endogenously expressed in a variety of tissues (source Human Atlas). To explore if endogenous BCA2 possesses antiviral activity, BCA2 was specifically depleted from 293T, HOS, Jurkat and 221T cells by shRNA. The targeted depletion of BCA2 in 293T and HOS cells, which do not express endogenous tetherin [15], resulted in a 3 to 5-fold increase in virus release, corroborating that BCA2 has antiviral activity even in the absence of tetherin. The depletion of BCA2 in T cell lines led to up to 15-fold increase in virus replication compared to shRNA-scrambled-treated cells. Since BCA2 impairs virus assembly and release, an increase in virus production will also be reflected by an increase in virus replication, since more virions are being generated, which can therefore infect more target cells.

In summary, here we show that BCA2 functions mainly as a tetherin-independent factor that leads to the ubiquitination of HIV-1 and other retroviral Gag proteins and, consequently, their lysosomal degradation. Therefore, BCA2 poses a significant barrier in virus assembly and release. Further work will determine the range of viruses susceptible to this E3 ligase, if viral pathogens have evolved countermeasures to BCA2, and explore if BCA2 is connected to immune signaling pathways.

## Materials and Methods

### Plasmid DNA constructs

#### (a) *BST2* expression constructs

Human *BST2* (*hBST2*) and rhesus macaque *BST2* allele *rBST2.1* were cloned as previously described [22], [29].

#### (b) BCA2 expression constructs

Human BCA2 was cloned from cDNA of PBMCs into the pcDNA5 expression vector using the unique restriction sites AflII and BamHI. HA-tagged versions of BCA2, as well as BCA2 deleted mutants, were originated by PCR manipulation and cloned into pcDNA5.

#### (c) HIV-1, SIV and MLV proviral clones

Full-length HIV-1 NL4-3 proviral DNA (pNL4-3) was obtained through the NIH AIDS Reagent Program, Division of AIDS, NIAID, NIH from Dr. Malcolm Martin, and HIV-1 NL4-3 Δ*vpu* was generated by site-directed mutagenesis as previously described [22]. Full-length clones for wild-type SIV and SIV Δ*nef* were constructed from the following clones based on SIV_mac_239; p239SpSp5', pSP72-239-3' and pSP72-239-3'Δ*nef* [46], [47]. Full-length Mo-MLV proviral DNA (pNCS) was obtained from Addgene, Cambridge, MA.

#### (d) Gag expression constructs

HIV-1 Gag (pGag-EGFP) was obtained through the NIH AIDS Reagent Program, Division of AIDS, NIAID, NIH from Dr. Marilyn Res. A codon-optimized version of SIV Gag was subcloned into pEGFP using the unique restriction sites SacII and BamHI to obtain the fusion protein SIV Gag-EGFP. Mutations in HIV-1 and SIV Gag were introduced by Quickchange site-directed mutagenesis according to the manufacturer guidelines (Stratagene, La Jolla, CA). Gag deleted mutants were originated by overlapping PCR and cloned into pEGFP.

#### (e) Dominant-negative mutants

A Rab7A-RFP construct was kindly provided by Dr. I-Chue Huang, University of Riverside, CA. The dominant negative mutant Rab7 N125I was generated by quickchange site-directed mutagenesis as described above (Stratagene, La Jolla, CA).

### Virus release assays

293T cells (5×10^4^) were co-transfected with wild-type or deleted mutants of HIV-1 NL4-3, SIV_mac_239 or Mo-MLV proviral DNA (100 ng) and either empty vectors, pcDNA3-hBST2, pcDNA3-rBST2, pcDNA5-HA-BCA2 or pcDNA5-HA-BCA2 mutants (100 ng). Differences in the amount of plasmid DNA in each transfection were offset by the addition of empty vector (100 ng), either pcDNA3 or pcDNA5. All transfections were performed in duplicate in 24-well plates using GenJet Lipid Transfection Reagents (SignaGen Laboratories, Gaithersburg, MD), and results were confirmed in at least three independent assays. Forty-eight hours post-transfection, the amount of virions released into the cell culture supernatant was measured by HIV-1 p24, SIV p27 antigen-capture ELISA (Advanced Bioscience Laboratories, Inc., Kensington, MD) or MuLV p30 core ELISA (Cell Biolabs, San Diego, CA), and virus release was expressed as the percentage of maximal virus release in the absence of BCA2 or tetherin, as previously described [22], [25], [29].

A similar approach was performed for assays with proteasomal and lysosomal inhibitors. Twenty-four hours post-transfection, the cell medium was changed and fresh medium was added containing either DMSO, ALLN (25 µM), clasto-Lactacystin β-lactone (20 µM), Chloroquine (0.1 nM), Leupeptin (20 µM), E64 (10 µM) or Pepstastin (20 µM). The accumulation of virus particles in the cell culture supernatant was assessed forty-eight hours post-transfection by HIV-1 p24 or SIV p27 antigen-capture ELISA, and virus release was expressed as the percentage of maximal release in the absence of HA-BCA2.

### Influenza infectivity

293T cells (3×10^5^) were seeded in 6-well plates. Twenty-four hours later, cells were transfected with increasing concentrations (0–1000 ng) of pcDNA5-HA-BCA2. Differences in DNA concentrations were offset by the addition of empty vector (pcDNA5). Twenty-four hours later, cells were infected with influenza H1N1 PR8 (kindly provided by Dr. Gack, Harvard Medical School) at a multiplicity of infection (MOI) of 3. Two days post-infection, the cell culture supernatant containing newly formed H1N1 virions was collected, and used for a second infectivity assay (1 ml of culture supernatant) on parental 293T cells, which were seeded the day before at 3x10^5^ cells per well in 6-well plates. Twenty-four hours after this second infection, cells were carefully detached using cell dissociation buffer (Sigma-Aldrich, St Louis, MO) and stained for the surface expression of the influenza hemagglutinin protein (HA). For this, a mouse monoclonal antibody anti-HA was used (Takara Bio, Japan) at a dilution of 1∶100, followed by washing steps with PBS and incubation with a secondary goat anti-mouse IgG PE-conjugated antibody (Sigma-Aldrich, St Louis, MO). After two washing steps with PBS, cells were fixed with 2% paraformaldehyde and analyzed by flow cytometry. Data were collected using a FACSCalibur flow cytometer (Becton Dickenson) and analyzed using FlowJo 8.8.7 software (TreesStar). Results were corroborated in two additional independent assays.

### Western blots

The whole cell lysates and the culture supernatants of transfected cells were analyzed by western blot, as previously described [22], [25], [29]. Forty-eight hours post-transfection, cell lysates were prepared by harvesting in 2x SDS sample buffer (Sigma-Aldrich, St Louis, MO). Virions were recovered from the cell culture supernatant by centrifugation at 14,000 rpm for 2 hours at 4°C, and resuspended in 2x SDS sample buffer. Samples were boiled for 5 minutes, and separated by electrophoresis on 8–12% SDS-polyacrylamide gels and transferred to polyvinylidine fluoride (PVDF) membranes using a Trans-Blot SD transfer cell (BioRad, Hercules, CA). The membranes were then blocked with 5% non-fat dry-milk in PBS containing 0.05% Tween-20 for 1 hour, and probed overnight at 4°C with one of the following primary antibodies. Endogenous BCA2 was detected with a rabbit polyclonal antibody against ZNF364 (abcam, Cambridge, MA) at a dilution of 1∶500. Tetherin/BST2 was detected with a mouse polyclonal antibody (abcam, Cambridge, MA) at a dilution of 1∶500. The HIV-1 and SIV Gag proteins p55 and p24 or p27, respectively, were detected with the mouse monoclonal antibody 183-H12-5C (AIDS Research and Reference Reagent Program, Division of AIDS, NIAID, NIH) at a dilution of 1∶1000. HIV-1 Nef protein was detected with rabbit antiserum (AIDS Research and Reference Reagent Program, Division of AIDS, NIAID, NIH) at 1∶500 dilution. HIV-1 Vpu was detected with rabbit antiserum (AIDS Research and Reference Reagent Program, Division of AIDS, NIAID, NIH) at 1∶500 dilution. SIV Nef was detected using the mouse monoclonal antibody 17.2 (AIDS Research and Reference Reagent Program, Division of AIDS, NIAID, NIH) at a dilution of 1∶1000. SIV Env was detected with the mouse monoclonal antibody KK41 (AIDS Research and Reference Reagent Program, Division of AIDS, NIAID, NIH) at a dilution of 1∶1000. Endogenous β-actin was detected with the mouse monoclonal antibody C4 (Chemicon, Billerica, MA) at a dilution of 1∶1000. HA-tagged BCA2 and other BCA2 mutants were detected with the HA-specific mouse monoclonal antibody HA.11 (Covance, Princeton, NJ) at a dilution of 1∶1000. The GFP fusion proteins HIV-1 Gag-GFP and SIV Gag-GFP were detected using either an anti-GFP mouse monoclonal antibody (Sigma-Aldrich, St Louis, MO) at a dilution of 1∶1000, a rabbit monoclonal antibody (abcam, Cambridge, MA) at a dilution of 1∶1000 or the mouse monoclonal anti-Gag (AIDS Research and Reference Reagent Program). In this case, a difference in the pattern of HIV-1 Gag-GFP and SIV Gag-GFP proteins is observed, since this antibody has more affinity for HIV-1 Gag and therefore, the higher molecular weight bands for SIV Gag are difficult to detect. After rinsing the PVDF membranes three times for 15 minutes in PBS 0.05% Tween-20, the blots were probed with an HRP-conjugated goat anti-mouse secondary antibody (Pierce, Rockford, IL) or goat anti-rabbit secondary antibody (abcam, Cambridge, MA) at a dilution of 1∶2000 for 1 hour at room temperature. The blots were then rinsed three more times in PBS 0.05% Tween-20, treated with SuperSignal West Femto Maximum Sensitivity substrate (Pierce, Rockford, IL), and imaged using a Fujifilm Image Reader LAS 3000 (Fujifilm Photo Film Co., Japan).

### Immunoprecipitation and ubiquitination assays

293T cells (6×10^5^ cells) were seeded in duplicate in 6-well plates. Twenty-four hours later, cells were co-transfected with constructs expressing wild-type and mutant forms HIV-1 Gag-GFP, SIV Gag-GFP (2 µg) along with wild-type or mutants constructs for HA-BCA2 or empty vector (pCDNA5) (2 µg). Similar assays were performed in the context of Gag proteins expressed from proviral DNA (HIV-1 NL4-3 and SIV_mac_239). Twenty-four hours later, cells were lysed with 400 µl of IP Lysis buffer (Thermo Scientific, Rockford, IL) and incubated on ice for 30 minutes. Lysates were transferred to 1.5 ml tubes and insoluble cell debris was removed by centrifugation at 8,000 x *g*. Cell lysate (100 µl) was set aside to confirm HA-BCA2 and Gag expression by western blot analysis, and the rest of the sample (300 µl) was used for immunoprecipitation. Samples for immunoprecipitation were incubated on a rotating platform for 1 hour at 4°C with 1 µg of the anti-HA mouse monoclonal antibody (Covance, Princeton, NJ), anti-GFP rabbit monoclonal antibody (abcam, Cambridge, MA) or mouse monoclonal antibody 183-H12-5C (AIDS Research and Reference Reagent Program, Division of AIDS, NIAID, NIH). Magnetic protein A sepharose beads (25 µl) (New England Biolabs, Ipswich, MA) were then added, and the incubation was continued overnight at 4°C. The beads were washed five times in IP Lysis buffer (500 µl) and boiled in 2x SDS sample buffer. Denatured proteins were separated on 8–12% SDS-polyacrylamide gels and transferred to PVDF membranes. The blots were probed with a mouse monoclonal antibody specific for Ubiquitin (Invitrogen, Grand Island, NY), a mouse monoclonal antibody to detect HA (Covance, Princeton, NJ), the mouse monoclonal antibody 183-H12-5C to detect Gag (AIDS Research and Reference Reagent Program, Division of AIDS, NIAID, NIH), or a rabbit monoclonal to detect GFP (abcam, Cambridge, MA) at a dilution of 1∶1000. Membranes were next probed with an HRP-conjugated goat anti-mouse antibody (Pierce, Rockford, IL), or goat anti-rabbit secondary antibody (abcam, Cambridge, MA) at a dilution of 1∶2000, developed in SuperSignal West Femto Maximum Sensitivity substrate and imaged using a Fujifilm Image Reader LAS 3000, as described above.

### shRNA-knockdown of endogenous BCA2

shRNA constructs to specifically deplete endogenous BCA2, as well as scrambled shRNA, were provided in lentiviral vectors (pLKO.1) through the Thermo Scientific TRC consortium (Broad Institute, MIT and Harvard). Each construct was designed to contain a hairpin consisting on a 21 base paired stem (sense and antisense strands), separated by a 6 base loop. The following BCA2-specific shRNAs were used for our depletion studies: TRCN0000004391 (referred hereafter as sh-BCA2-1), TRCN0000004392 (referred hereafter as sh-BCA2-2), TRCN0000004393 (referred hereafter as sh-BCA2-3), TRCN0000004394 (referred hereafter as sh-BCA2-4) and TRCN0000010871 (referred hereafter as sh-BCA2-5). The best knockdowns were achieved with constructs sh-BCA2-1, sh-BCA2-3 and sh-BCA2-4 (scores of 4.95, 15 and 10.8, respectively). The targeted depletion of BCA2 was afforded by transient transfection on 293T and HOS cells, according to the manufacturer instructions (Thermo Scientific, Lafayette, CO). For replication assays in Jurkat and 221T cells, VSV-G pseudotyped lentiviral particles were produced to transduce each shRNA construct. The particles were generated by transient transfection on 293T cells using psPAX2 packaging plasmid and pMD2.G envelope expressing plasmid according to the manufacturer's instructions (Thermo Scientific, Lafayette, CO). Efficient knockdown of BCA2 was assessed by western blotting after the second round of transduction from 10^6^ cells, and compared to the expression of β-actin.

### Virus replication in BCA2-depleted cells

Human Jurkat CD4^+^ T cells and rhesus macaque 221T cells [40], were transduced with VSV-G pseudotyped lentiviral particles carrying scrambled shRNA or BCA2-specific shRNAs four days prior to HIV-1 or SIV infection. After 3 hours of incubation at 37°C, cells were washed and resuspended in 5 ml of R10 or R20 + IL-2 (100 U) medium, respectively. Forty-eight hours later, the cells were subjected to a second round of lentiviral transduction. Two days later, we proceeded with the infectivity assays. First, 10^6^ cells were set aside for knockdown verification by western blot. 10^6^ Jurkat T cells or 221 T cells were infected with 20 ng of p24 HIV-1 NL4-3 or 20 ng of p27 of SIV_mac_239, respectively, and incubated for 3 hours at 37°C. Next, cells were washed three times and resuspended in 5 ml of R10 or R20 + IL-2. Virus replication was monitored at selected time points by p24/p27 antigen-capture ELISA of the culture supernatant.

### Confocal microscopy

293T cells (2×10^4^ cells in a 8-well slide) were transfected with pcDNA5-HA-BCA2 and either pHIVGag-EGFP, pSIVGag-EGFP expression constructs, HIV-1 NL4-3 or SIV_mac_239 proviral DNA. Twenty-four hours later, cells were washed and fixed for 10 minutes in acetone/methanol and blocked for 20–60 minutes with 100 mM glycine diluted in 10% normal goat serum in PBS with 0.2% fish skin gelatin, 0.1% Triton x100 and 0.02% sodium azide (10% NGS-PBS-FSG-Tx100-NaN_3_). The cells were then washed three times in 10% NGS-PBS-FSG-Tx100-NaN_3_, and stained. Gag expression was tracked by GFP fluorescence. The mouse monoclonal antibody to HA (IgG_1_; Covance, Princeton, NJ) was used at a dilution of 1∶250 to stain for HA-BCA2. The cells were subsequently stained with an Alexa-568-conjugated goat anti-mouse secondary antibody specific for IgG_1_ (Invitrogen, Grand Island, NY) (1∶1000), and with TO-PRO3 (Invitrogen) (1∶5000) to visualize cell nuclei. In the case of transfections with proviral DNA, Gag expression was tracked by staining for CA (p24 or p27) with the mouse monoclonal antibody 183-H12-5C (IgG_1_; AIDS Research and Reference Reagent Program). A mouse monoclonal antibody to HA (IgG_2a_) was used to stain HA-BCA2 (Santa Cruz Biotechnologies, Santa Cruz, CA). Next, a secondary Alexa-488-conjugated goat anti-mouse IgG_1_ and an Alexa-633-conjugated goat anti-mouse IgG_2a_ were used to detect Gag and HA-BCA2, respectively. To stain intracellular compartments, rabbit polyclonal antibodies specific for TGN46 (Sigma-Aldrich, St Louis, MO), CD63 (Santa Cruz Biotechnology, Santa Cruz, CA) and LAMP-1 (abcam, Cambridge, MA) were used at a dilution of 1∶50. Next, an Alexa-568 goat anti-rabbit (Invitrogen, Grand Island, NY) was used to detect these cellular markers. After staining, the slides were washed and mounted on with antiquenching mounting-medium (Vector Laboratories, Inc.). Images were acquired using a Leica TCS SP5 II confocal microscope.

## Supporting Information

Figure S1 **The BCA2-related defect in Gag expression is BCA2-specific.** 293T cells were co-transfected with HIV-1 NL4-3 (A) or SIV_mac_239 (B) proviral DNA and constructs coding for CD8-STOP, HA-BCA2 or Dyn2-GFP. Virus release was measured 48 hours post-transfection as previously described. (C) The cell lysates of these transfected cells were analyzed by western blot for Gag and β-actin expression. Error bars represent standard deviation of independent experiments. (D) Titration curve of HA-BCA2. Membranes were probed with a rabbit polyclonal antibody against BCA2 to control for any effects of protein overexpression on Gag levels.
(TIF)

Figure S2 **BCA2 reduces virus release, and therefore, the amount of infectious particles.** (A) The infectivity of HIV-1 and SIV virions generated by transient transfection from parental 293T cells or HA-BCA2-expressing 293T cells was evaluated on GHOST X4/R5 cells by determining the GFP^+^ infected cells, and calculating the relative infectivity. (B) Cell lysates (WCL) and virions present in the culture supernatant of parental and HA-BCA2-expressing 293T cells were analyzed by western blot. Error bars represent standard deviation of independent experiments.
(TIF)

Figure S3 **BCA2 interferes with Mo-MLV particle release.** (A) 293T cells were co-transfected with Mo-MLV proviral DNA and expression vectors coding for human tetherin (hBST2), HA-BCA2 or the ligase-dead BCA2 mutant (ΔRing BCA2). Virus release was measured 48 hours post-transfection by MuLV p30 ELISA, and expressed as the maximal release in the absence of tetherin or HA-BCA2. (B) Cell lysates (WCL) and virions were analyzed by western blot for HA-BCA2, ΔRing BCA2 and tetherin expression, as well as Gag and p30. Error bars represent standard deviation of independent transfections.
(TIF)

Figure S4 **BCA2 binds to the Matrix region of HIV-1 Gag.** 293T cells were co-transfected with constructs coding for HA-BCA2, HIV-1 Gag-GFP or deleted forms of HIV-1 Gag (see Figure 4B). Twenty-four hours later, cell lysates were immunoprecipitated with a mouse monoclonal antibody anti-HA and membranes were developed with a rabbit polyclonal anti-GFP. Lysates (WCL) were also analyzed by western blotting to check the input levels of HA-BCA2, Gag-GFP constructs and β-actin. V: empty vector.
(TIF)

Figure S5 **Effects of proteasomal and lysosomal inhibitors on BCA2 activity. (**A) The effect of ALLN on the antiviral activity of BCA2 was explored by knocking down endogenous BCA2 in 293T cells treated with either DMSO or ALLN (25 µM), and by analyzing the expression levels of HIV-1 Gag in cells and p24 in virions. (B) Differences in the levels of Gag expression were quantified by ImageJ64 software for cells treated with DMSO (white) or ALLN (black), and the relative Gag expression was calculated. (C) To determine if the increase in virus released observed in HA-BCA2^+^ cells treated with lysosomal inhibitors is due to the specific inhibition of BCA2, the effects of these drugs on virus release were evaluated in parental 293T cells and HA-BCA2-expressing cells. Virus release was measured by HIV-1 p24 antigen-capture ELISA and expressed as the percentage of maximal release, as described in the material and methods section. (D) To evaluate the role of Rab7 in the mechanism of restriction by BCA2, virus release assays were performed in the presence of a dominant-negative mutant of Rab7A (Rab7 N125I). Virus release was measured by HIV-1 p24 and SIV p27 antigen-capture ELISA and expressed as the percentage of maximal release in the absence of HA-BCA2. Error bars represent the mean and standard deviation of independent experiments.
(TIF)

Figure S6 **BCA2 leads to the accumulation of HIV-1 Gag to TGN46-negative compartments.** 293T cells were co-transfected with constructs coding for HIV-1 NL4-3 proviral DNA and HA-BCA2. Cells were stained for p24 (green), HA-BCA2 (blue) and the cellular marker TGN46 (red). White scale bar corresponds to 10 µm.
(TIF)
