# Supplementary figures and images for: BCA2/Rabring7 Targets HIV-1 Gag for Lysosomal Degradation in a Tetherin-Independent Manner
Source: PLoS Pathog. 2014 May 22;10(5):e1004151. doi: 10.1371/journal.ppat.1004151 (PMC4031200; doi:10.1371/journal.ppat.1004151)

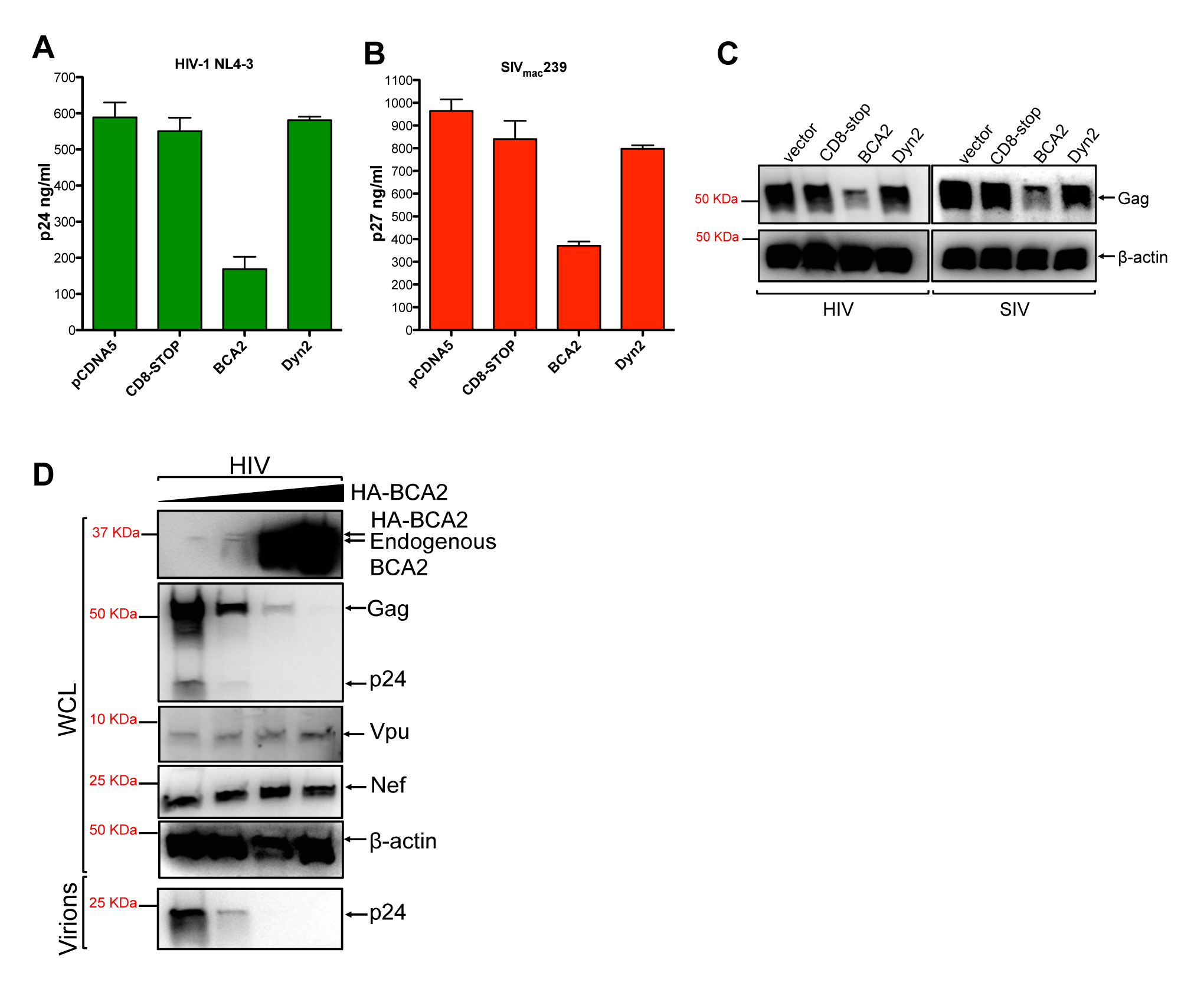

Supplement: Figure S1 — The BCA2-related defect in Gag expression is BCA2-specific. 293T cells were co-transfected with HIV-1 NL4-3 (A) or SIVmac239 (B) proviral DNA and constructs coding for CD8-STOP, HA-BCA2 or Dyn2-GFP. Virus release was measured 48 hours post-transfection as previously described. (C) The cell lysates of these transfected cells were analyzed by western blot for Gag and β-actin expression. Error bars represent standard deviation of independent experiments. (D) Titration curve of HA-BCA2. Membranes were probed with a rabbit polyclonal antibody against BCA2 to control for any effects of protein overexpression on Gag levels. (TIF) [file ppat.1004151.s001.tif]

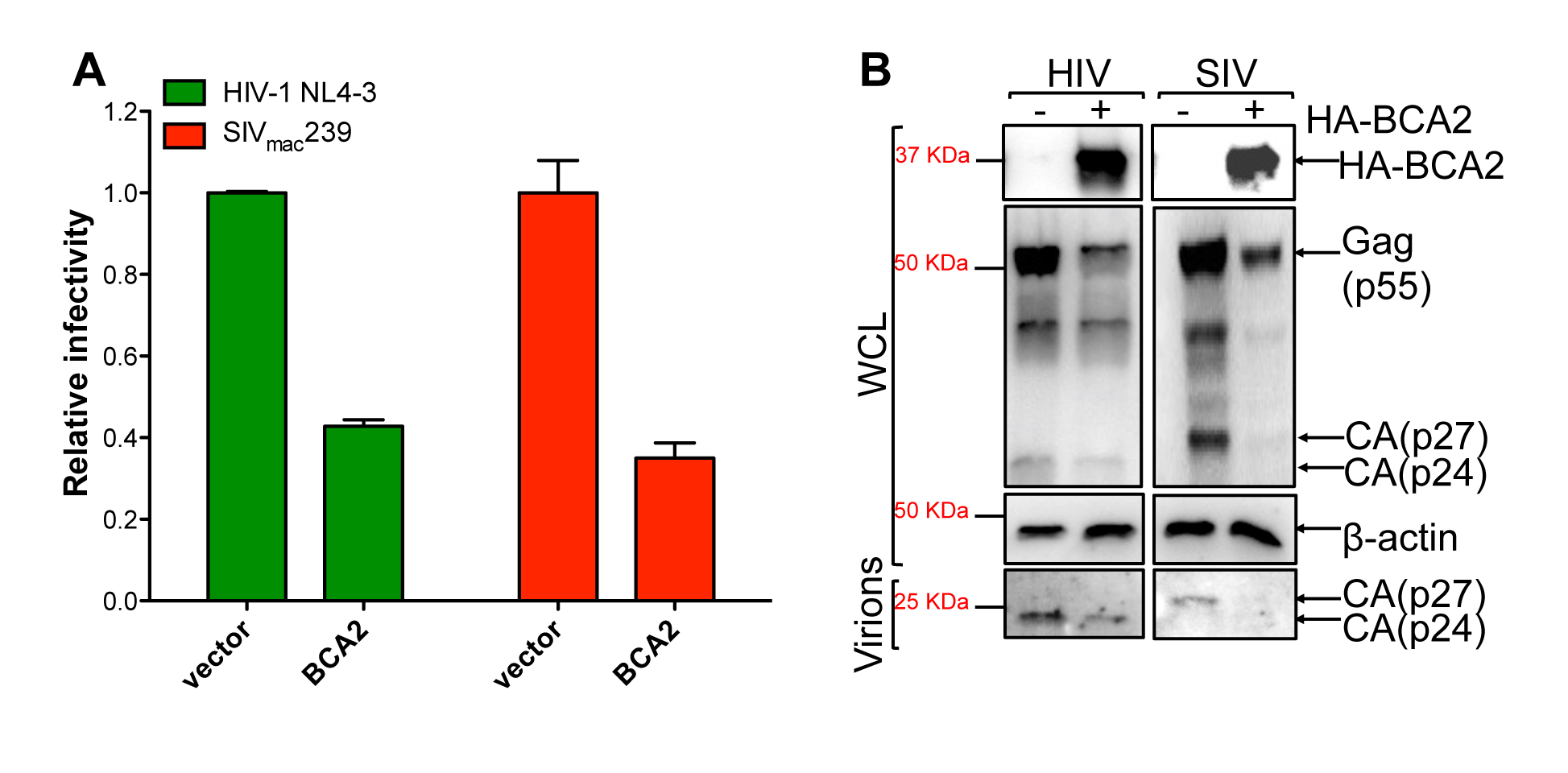

Supplement: Figure S2 — BCA2 reduces virus release, and therefore, the amount of infectious particles. (A) The infectivity of HIV-1 and SIV virions generated by transient transfection from parental 293T cells or HA-BCA2-expressing 293T cells was evaluated on GHOST X4/R5 cells by determining the GFP+ infected cells, and calculating the relative infectivity. (B) Cell lysates (WCL) and virions present in the culture supernatant of parental and HA-BCA2-expressing 293T cells were analyzed by western blot. Error bars represent standard deviation of independent experiments. (TIF) [file ppat.1004151.s002.tif]

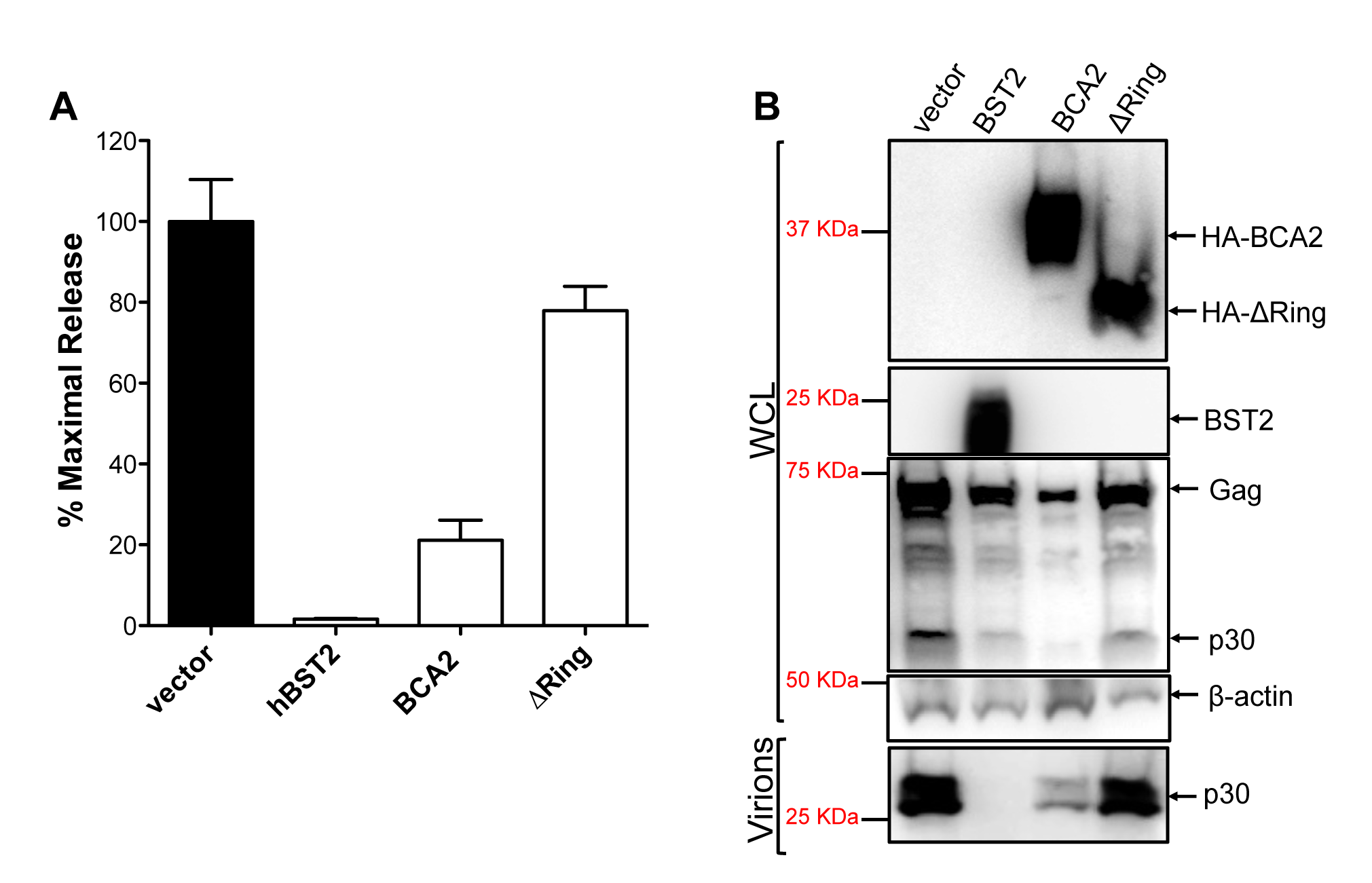

Supplement: Figure S3 — BCA2 interferes with Mo-MLV particle release. (A) 293T cells were co-transfected with Mo-MLV proviral DNA and expression vectors coding for human tetherin (hBST2), HA-BCA2 or the ligase-dead BCA2 mutant (ΔRing BCA2). Virus release was measured 48 hours post-transfection by MuLV p30 ELISA, and expressed as the maximal release in the absence of tetherin or HA-BCA2. (B) Cell lysates (WCL) and virions were analyzed by western blot for HA-BCA2, ΔRing BCA2 and tetherin expression, as well as Gag and p30. Error bars represent standard deviation of independent transfections. (TIF) [file ppat.1004151.s003.tif]

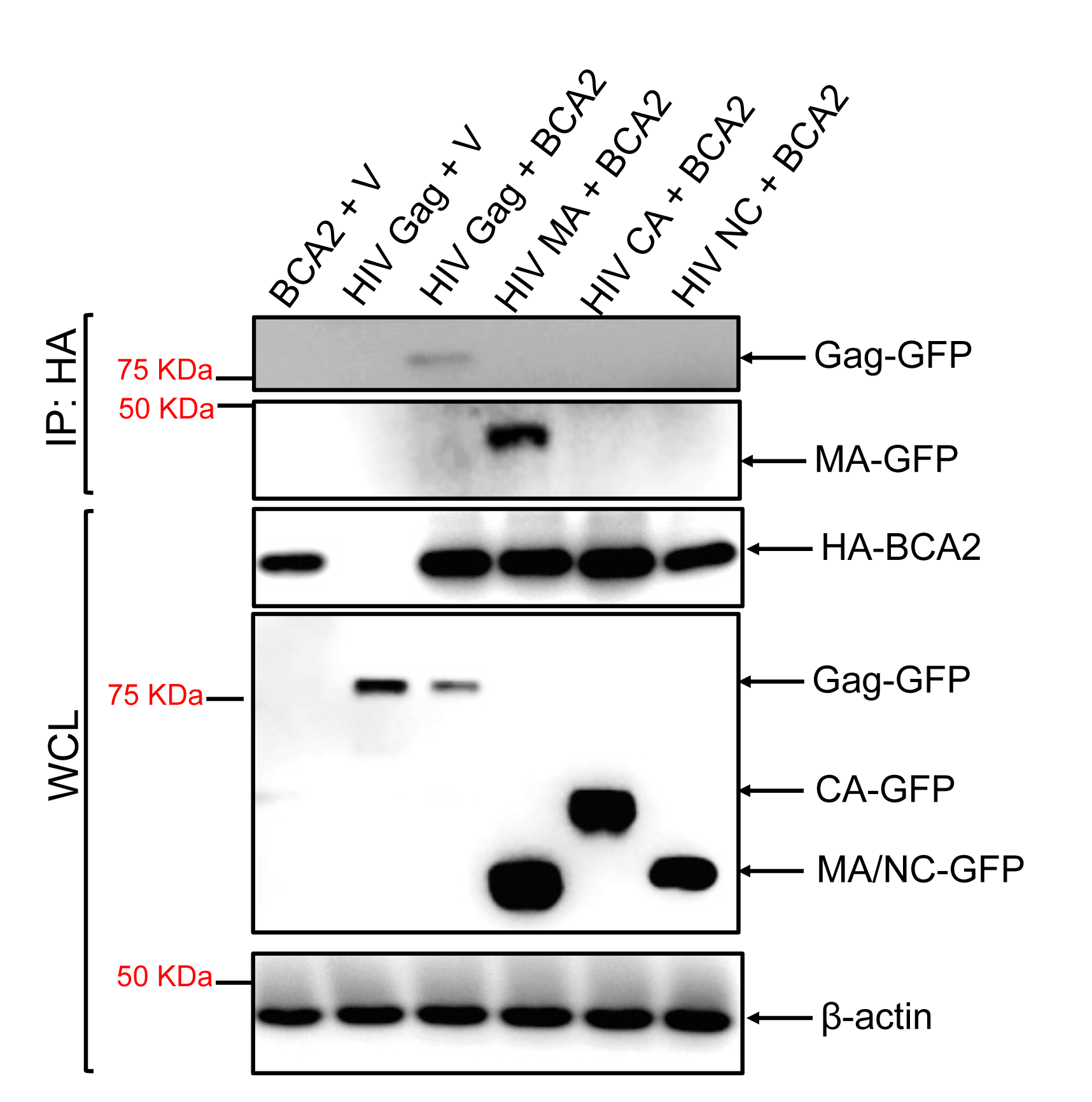

Supplement: Figure S4 — BCA2 binds to the Matrix region of HIV-1 Gag. 293T cells were co-transfected with constructs coding for HA-BCA2, HIV-1 Gag-GFP or deleted forms of HIV-1 Gag (see Figure 4B). Twenty-four hours later, cell lysates were immunoprecipitated with a mouse monoclonal antibody anti-HA and membranes were developed with a rabbit polyclonal anti-GFP. Lysates (WCL) were also analyzed by western blotting to check the input levels of HA-BCA2, Gag-GFP constructs and β-actin. V: empty vector. (TIF) [file ppat.1004151.s004.tif]

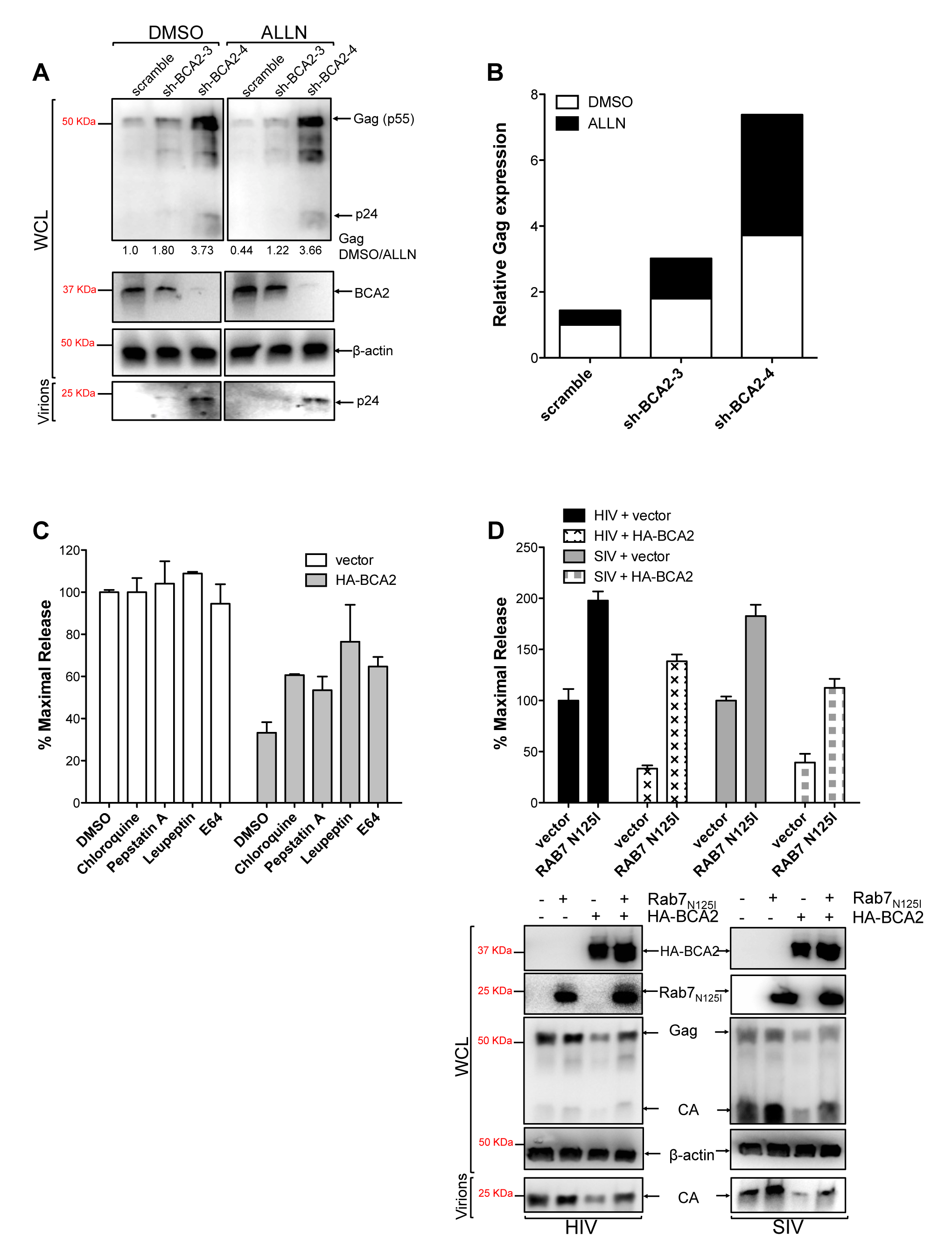

Supplement: Figure S5 — Effects of proteasomal and lysosomal inhibitors on BCA2 activity. (A) The effect of ALLN on the antiviral activity of BCA2 was explored by knocking down endogenous BCA2 in 293T cells treated with either DMSO or ALLN (25 µM), and by analyzing the expression levels of HIV-1 Gag in cells and p24 in virions. (B) Differences in the levels of Gag expression were quantified by ImageJ64 software for cells treated with DMSO (white) or ALLN (black), and the relative Gag expression was calculated. (C) To determine if the increase in virus released observed in HA-BCA2+ cells treated with lysosomal inhibitors is due to the specific inhibition of BCA2, the effects of these drugs on virus release were evaluated in parental 293T cells and HA-BCA2-expressing cells. Virus release was measured by HIV-1 p24 antigen-capture ELISA and expressed as the percentage of maximal release, as described in the material and methods section. (D) To evaluate the role of Rab7 in the mechanism of restriction by BCA2, virus release assays were performed in the presence of a dominant-negative mutant of Rab7A (Rab7 N125I). Virus release was measured by HIV-1 p24 and SIV p27 antigen-capture ELISA and expressed as the percentage of maximal release in the absence of HA-BCA2. Error bars represent the mean and standard deviation of independent experiments. (TIF) [file ppat.1004151.s005.tif]

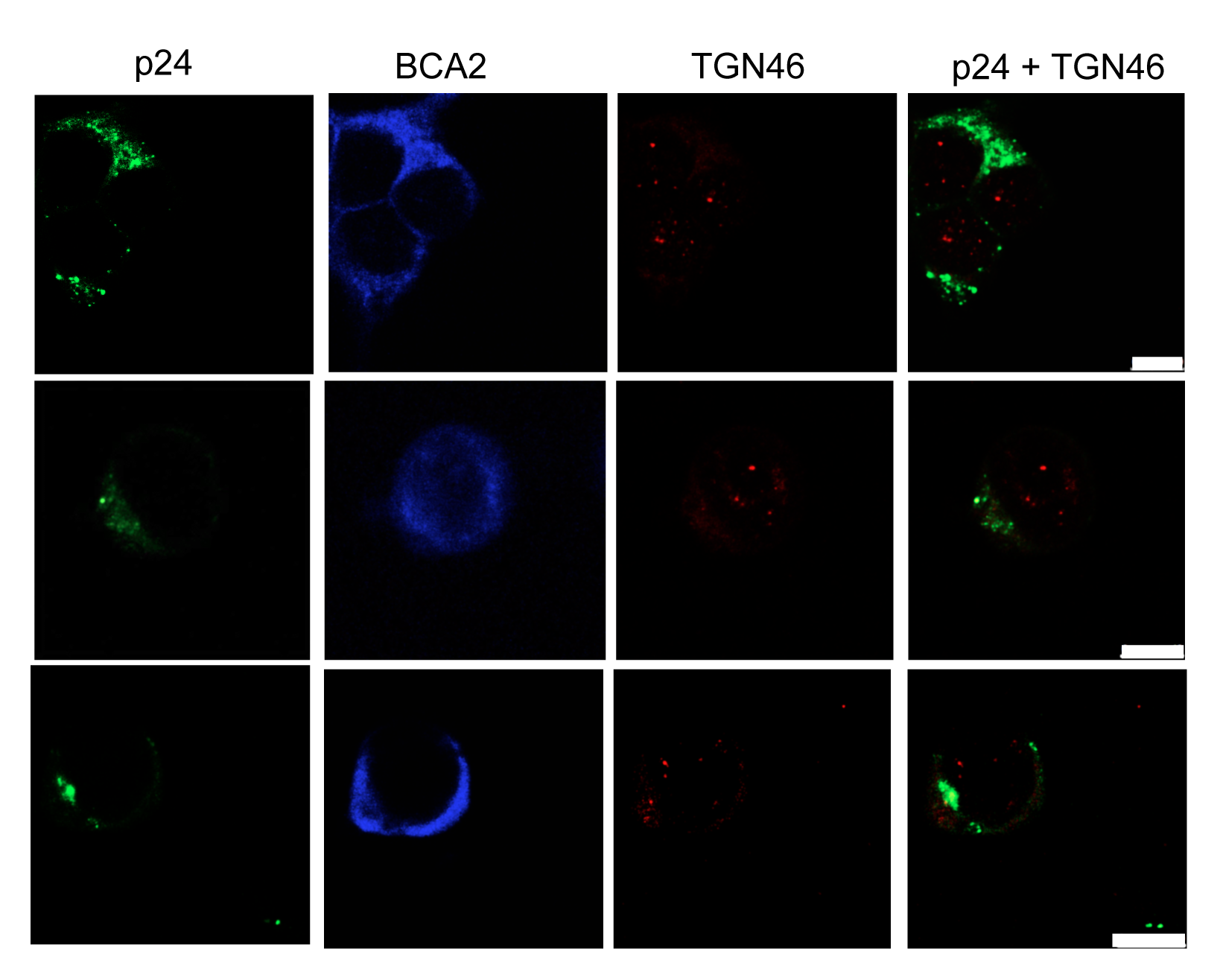

Supplement: Figure S6 — BCA2 leads to the accumulation of HIV-1 Gag to TGN46-negative compartments. 293T cells were co-transfected with constructs coding for HIV-1 NL4-3 proviral DNA and HA-BCA2. Cells were stained for p24 (green), HA-BCA2 (blue) and the cellular marker TGN46 (red). White scale bar corresponds to 10 µm. (TIF) [file ppat.1004151.s006.tif]
